# A Mass Spectrometry-Based Approach for Characterization of Red, Blue, and Purple Natural Dyes

**DOI:** 10.3390/molecules25143223

**Published:** 2020-07-15

**Authors:** Katarzyna Lech, Emilia Fornal

**Affiliations:** 1 Faculty of Chemistry, Warsaw University of Technology, Noakowskiego 3, 00-664 Warsaw, Poland; 2 Department of Pathophysiology, Medical University of Lublin, Jaczewskiego 8b, 20-090 Lublin, Poland; emilia.fornal@umlub.pl

**Keywords:** natural dyes, orchil, sandalwood, brazilwood, tandem mass spectrometry, textile

## Abstract

Effective analytical approaches for the identification of natural dyes in historical textiles are mainly based on high-performance liquid chromatography coupled with spectrophotometric detection and tandem mass spectrometric detection with electrospray ionization (HPLC-UV-Vis-ESI MS/MS). Due to the wide variety of dyes, the developed method should include an adequate number of reference color compounds, but not all of them are commercially available. Thus, the present study was focused on extending of the universal analytical HPLC-UV-Vis-ESI MS/MS approach to commercially unavailable markers of red, purple, and blue dyes. In the present study, HPLC-UV-Vis-ESI MS/MS was used to characterize the colorants in ten natural dyes (American cochineal, brazilwood, indigo, kermes, lac dye, logwood, madder, orchil, Polish cochineal, and sandalwood) and, hence, to extend the analytical method for the identification of natural dyes used in historical objects to new compounds. Dye markers were identified mostly on the basis of triple quadrupole MS/MS spectra. In consequence, the HPLC-UV-Vis-ESI MS/MS method with dynamic multiple reaction monitoring (dMRM) was extended to the next 49 commercially unavailable colorants (anthraquinones and flavonoids) in negative ion mode and to 11 (indigoids and orceins) in positive ion mode. These include protosappanin B, protosappanin E, erythrolaccin, deoxyerythrolaccin, nordamnacanthal, lucidin, santalin A, santalin B, santarubin A, and many others. Moreover, high-resolution QToF MS data led to the establishment of the complex fragmentation pathways of α-, β-, and γ- aminoorceins, hydroxyorceins, and aminoorceinimines extracted from wool dyed with *Roccella tinctoria* DC. The developed approach has been tested in the identification of natural dyes used in 223 red, purple, and blue fibers from 15th- to 17th-century silk textiles. These European and Near Eastern textiles have been used in vestments from the collections of twenty Krakow churches.

## 1. Introduction

High-performance liquid chromatography (HPLC) is the most common technique used for the separation of colorants present in natural dyes and historical textiles [1,2,3]. Since the amount of available research material is usually limited, it is extremely important to use a sensitive detector that is both selective and versatile for a variety of coloring compounds. Thus, the best results are acquired using both spectrophotometric (UV-Vis) and mass spectrometric (MS) detection together [4,5,6,7,8,9,10,11,12,13]; however, they are used individually as well [14,15,16,17,18,19,20,21,22].

The best separations to date have been achieved using reversed phase columns, mostly C18. However, since colorants have extended systems of conjugated double bonds, it seems reasonable to use columns with phenyl-type stationary phases capable of exploiting pi–pi interactions to achieve separation. The correct identification of a dye requires, at first, the true positive identification of analytes, which has to be based on the comparison of not only their retention times but also their other physicochemical properties (such as *m*/*z* values and characteristic absorption) with those of reference compounds determined under identical experimental conditions. Therefore, it is important to prepare a wide/extensive database of markers covering the largest possible number of dyes.

Unfortunately, some of the colorants are commercially unavailable, and thus, their identification can be troublesome. Therefore, a tandem mass spectrometer (MS/MS) as an HPLC detector turns out to be extremely useful. Since the fragmentation pathways of colorants within classes are rather similar, some ions may have diagnostic functions [12,23,24,25,26]. They can provide structural information about eluted compounds [27,28,29], providing a basis for their identification. Moreover, a tandem mass spectrometer with a triple quadrupole offers multiple reaction monitoring (MRM), which is particularly useful for the specific analysis of target compounds in complex mixtures.

Most HPLC methods published to date have been dedicated to identifying specific groups of colorants, such as anthraquinones from madder and scale insects [7,8,9,10,11,14,17,18,19,20,21,22,30,31] or indigoids [5,7,8,9,11,12,13,14,15,16,20,21,32], whereas only one was about orchil colorants [33,34,35]. However, it appears that there are no reports devoted to sandalwood colorants. Additionally, not all brazilwood markers have been identified yet, despite recent successes in this field [36]. Furthermore, to the best of our knowledge, there is no publication on a universal analytical approach discussing dozens of colorants, as opposed to being limited to just selected and the most popular compounds or their groups.

The present study was focused on the development of a comprehensive analytical approach for the identification of natural dyes in historical objects, antiques, and works of art. To do so, research was carried out using high-performance liquid chromatography coupled with spectrophotometric detection and tandem mass spectrometric detection with electrospray ionization (HPLC-UV-Vis-ESI MS/MS) and quadrupole time-of-flight mass spectrometry (QToF MS), which resulted in the determination of commercially unavailable markers present in ten red, purple, and blue dyes (American cochineal, brazilwood, indigo, kermes, lac dye, logwood, madder, orchil, sandalwood, and Polish cochineal). It led, among that of others, to the identification of α-, β-, and γ-aminoorceins, hydroxyorceins, and aminoorceinimines in wool dyed with *Roccella tinctoria* DC and, thanks to high-resolution data, to the specification of their complex fragmentation pathways. As a consequence, the new markers were introduced to the HPLC-UV-Vis-ESI MS/MS developed method. This became a base for creating an analytical approach that combines an extraction protocol and detection parameters (UV-Vis and MS) depending on sample color and, consequently, on pending analytes. This approach was subsequently applied to analyze 223 thread samples from silk textiles dated from the 15th to 17th century and used in vestments from the collections of twenty Krakow churches. The obtained results—together with already-published data on yellow, brown, and green threads [37,38]—have completed the picture of natural dyes used in the most valuable 15th- to 17th-century textiles of European and Near Eastern origin.

## 2. Results and Discussion

The previously developed HPLC-UV-Vis-MS/MS method (incorporating commercially available standards and markers of yellow, orange, and brown dyes) [37] was extended to colorants present in another ten dyes (American cochineal, brazilwood, indigo, kermes, lac dye, logwood, madder, orchil, sandalwood, and Polish cochineal). The compounds were identified according to the MS/MS spectra acquired for various collision energies (CEs). The most intense precursor and product ion pairs (transitions) of the identified dye markers were used to develop the final method in dynamic multiple reaction monitoring (dMRM) mode, which provides superior sensitivity and selectivity for targeted compounds in complex samples. In a dMRM experiment, analytes are only monitored while they are being eluted from the LC (during the retention window), so the MS duty cycle is not wasted by monitoring them when they are not expected, which maximizes the detection capability of the MS. The first quadrupole is set to pass a desired precursor ion, the second quadrupole is used as a collision cell to fragment that precursor ion, and the third quadrupole is set to monitor a specific fragment ion. The new colorants and their MS transitions are shown in detail in Table 1. Consequently, the method was applied to investigate dyes used in 15th- to 17th-century textiles.

### 2.1. Dyes

Colorants extracted from indigo as well as from wool fibers dyed with nine other dyes were identified using an ESI MS/MS detector preceded by HPLC with a phenyl column. Full scan analysis and the subsequent MS/MS fragmentations of the predominant quasi-molecular ions were used to obtain information about the molecular weights of the colorants and for the structural evaluation of the sugar moieties, aglycones, and unglycosylated compounds.

#### 2.1.1. Indigo

**Figure 1:**
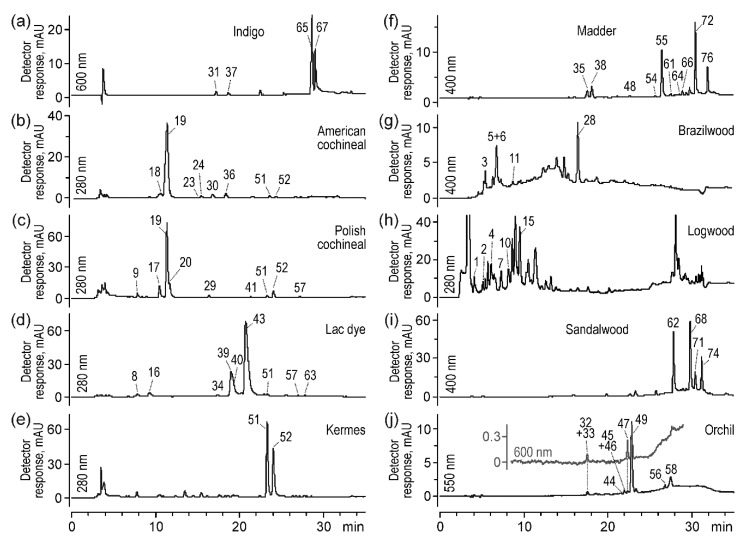
Chromatogram of Polynesian indigo (**a**), American cochineal (**b**), Polish cochineal (**c**), lac dye (**d**), kermes (**e**), madder (**f**), brazilwood (**g**), logwood (**h**), sandalwood (**i**), and orchil (**j**) extracts acquired by UV-Vis detector; peak numbers are decoded in Table 1.

Chromatograms acquired for a DMSO extract of indigo showed two main peaks that corresponded to indigotin (***65***) ([M + H]^+^ at *m*/*z* 263) and indirubin (***67***) ([M + H]^+^ at *m*/*z* 263). They were observed with a spectrophotometer at 280, 550, and 600 nm and with an MS detector in positive ion mode. In addition, two small peaks were found at *t_R_* 17.2 and 18.4 min (Figure 1a). The MS investigation of these two compounds suggested that they might be isomers. Firstly, the even *m*/*z* values of both their [M + H]^+^ ions were 262, which indicated an odd number of nitrogen atoms in their molecules. Secondly, their MS/MS spectra were very similar to each other although not identical (Supplementary Materials, Figure S1). Apart from the ions at *m*/*z* 245, 235, 219, and 190 present in both mass spectra, the first compound (*t_R_* 17.2 min) showed an intense signal at *m*/*z* 120, whereas the second one (*t_R_* 18.4 min) showed such at *m*/*z* 131. These MS/MS spectra almost completely coincided with those acquired for indigotin and indirubin, especially for *m*/*z* values above 150, indicating similarities between their structures too. Looking at the data taken together, the chemical formula of both compounds was defined as C_16_H_11_N_3_O; however, their molecular structures could not be determined. Nevertheless, it seemed that these compounds (called indigoid compound A (***31***) and B (***37***)) would not be crucial for identifying indigo in historical objects.

#### 2.1.2. Scale Insect Dyes

Colorants present in American and Polish cochineals were the subject of earlier detailed studies [24,25]. On this basis, apart from carminic and kermesic acids (***52***), flavokermesic acid (***51***), dcII (***18***), dcIV (***30***), dcVII (***36***), dc*O*fka (***23***), dc3 (***22***), and dc4 (***25***) (Figure 1b) as well as pp6 (***17***), pp7 (***20***), and deoxyerythrolaccin (***57***) (Figure 1c) were included in the presented method, as they were previously recommended as American and Polish cochineal markers, respectively.

Chromatographic and spectrometric data acquired for the lac dye extract proved the presence of several laccacic and xantholaccaic acids [39] (Figure 1d). Since they are animal-origin oxidized derivatives of kermesic and flavokermesic acids substituted at the C-7 position by a large functional group (Supplementary Materials, Figure S2)—that is, by *N*-acetyltyramine (in laccaic acid A and xantholaccaic acid A), tyrosal (laccaic acid B and xantholaccaic acid B), tyrosine (laccaic acid C), or tyramine (laccaic acid E)—their [M − H]^−^ ions showed almost identical fragmentation pathways. The two most intense signals in each MS/MS spectrum (Supplementary Materials, Figure S3) corresponded to the loss of one or two CO_2_ molecules from carboxyl groups, whereas the next one was a result of the further loss of a H_2_O molecule.

**Figure 2:**
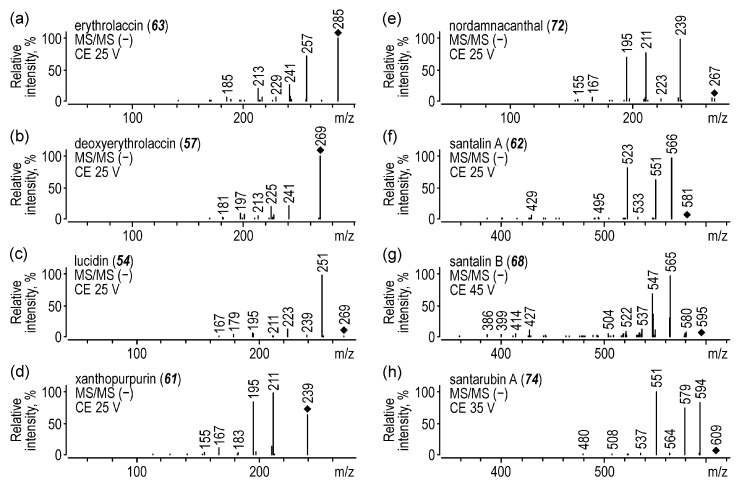
MS/MS spectra of (**a**) erythrolaccin, (**b**) deoxyerythrolaccin, (**c**) lucidin, (**d**) xanthopurpurin, (**e**) nordamnacanthal, (**f**) santalin A, (**g**) santalin B, and (**h**) santarubin A acquired in negative ion mode.

Apart from laccaic and xantholaccaic acids, the lac dye extract also contained kermesic acid (***52***) ([M − H]^−^ at *m*/*z* 329) and flavokermesic acid (***51***) ([M − H]^−^ at *m*/*z* 313), as well as their decarboxylated derivatives, erythrolaccin (***63***) ([M − H]^−^ at *m*/*z* 285) and deoxyerythrolaccin (***57***) ([M − H]^−^ at *m*/*z* 269). Their fragmentation pathways were also similar to each other. The signals at *m*/*z* 257, 241, 229, and 213 for erythrolaccin (Figure 2a) and at *m*/*z* 241, 225, 213, and 197 for deoxyerythrolaccin (Figure 2b) corresponded to the [M−H−CO]^−^, [M−H−CO_2_]^−^, [M−H−2CO]^−^, and [M−H−CO_2_−CO]^−^ ions, respectively.

Chromatograms of the kermes extract (Figure 1e) showed two main peaks of kermesic acid (***52***) and flavokermesic acid (***51***) as well as traces of deoxyerythrolaccin (***57***). Next, two small peaks eluted at 10.5 and 16.3 min were identified to be pp6 (***17***) (flavokermesic acid *O*-hexoside, *m*/*z* 475–431, 269) and pp9 (***27***) (kermesic acid *O*-hexoside, *m*/*z* 491–447, 284), respectively. Their MS/MS spectra showed the same fragmentation pattern, according to which the first signals corresponded to the [M−H−CO_2_]^−^ ions, and the next ones were formed by the subsequent loss of hexose moieties ([M−H−CO_2_−Hex]^−^ or [M−H−CO_2_−Hex]^−^).

#### 2.1.3. Madder

Chromatograms of the madder extract acquired by the spectrophotometric detector showed several peaks. The most intense peak belonged to alizarin (***55***), but purpurin (***64***) and rubiadin (***66***) were also found (Figure 1f). These colorants were identified by comparison with their standards, while others required the analysis of MS/MS spectra.

A compound eluted just before alizarin and observed in the chromatograms acquired at 280 and 400 nm was identified to be lucidin (***54***). MS/MS spectra of its [M − H]^−^ ion at *m*/*z* 269 (Figure 2c) showed three intense signals at *m*/*z* 251, 223, and 295 corresponding to the loss of H_2_O from a terminal aliphatic hydroxyl group and to the further elimination of one or two CO molecules, respectively. Compounds eluted at *t_R_* 27.5 and 30.5 min were identified to be xanthopurpurin (***61***) ([M − H]^−^ at *m*/*z* 239, Figure 2d) and nordamnacanthal (***72***) ([M − H]^−^ at *m*/*z* 267, Figure 2e), respectively. They differ from each other only by the presence of an aldehyde group at the C-2 position of nordamnacanthal that fragmented in the first place, giving the [M−H−CO]^−^ ion at *m*/*z* 239. Apart from that, all the other signals in both spectra were the same; the ions at *m*/*z* 211, 195, and 167 were formed by the further loss of CO, CO_2_, or both of these molecules together, respectively. The MS/MS spectra of xanthopurpurin and nordamnacanthal were identical to those ones found in the literature [40,41]. Similar congruence was found for the absorbance spectra of other identified compounds.

Another peak was observed at 32.1 min in the chromatograms acquired with spectrophotometric detection at visible range. This compound (coded as rt1) has not been reported to date. Simple MS/MS spectra of its [M − H]^−^ ion at *m*/*z* 491 included only two significant signals (Supplementary Materials, Figure S4), the first one at *m*/*z* 239 and the second one at *m*/*z* 251. It led to the assumption that the colorant was an anthraquinone dimer, probably composed of one alizarin molecule and one lucidin molecule (***76***).

Apart from the anthraquinones described above, some of their glycosidic derivatives were found, that is, lucidin *O*-primeveroside (***35***) ([M − H]^−^ at *m*/*z* 563), ruberthyric acid (***38***) ([M − H]^−^ at *m*/*z* 533), and rubiadin *O*-primeveroside (***48***) ([M − H]^−^ at *m*/*z* 547). They were identified mainly thanks to the characteristic loss of 294 Da corresponding to the primeverosyl moiety, and by the comparison of their further fragments with those observed for lucidin (***54***), alizarin (***55***), and rubiadin (***66***) (Supplementary Materials, Figure S5).

#### 2.1.4. Brazilwood

According to the literature, brazilwood contained mainly brazilin (***6***) and brazilein (***3***). Peaks of these two compounds were found at *t_R_* 7.1 and 5.5 min, respectively, in the chromatogram of the brazilwood extract (Figure 1g). Their identification was based on MS/MS spectra.

The fragmentation of brazilin-type neoflavonoids has been discussed in only one publication to date [42], but the considerations have been devoted to one particular fusion, and they have not included the complete pathway, especially for brazilin. Nevertheless, the presented data were helpful in the identification of both neoflavonoids.

**Figure 3:**
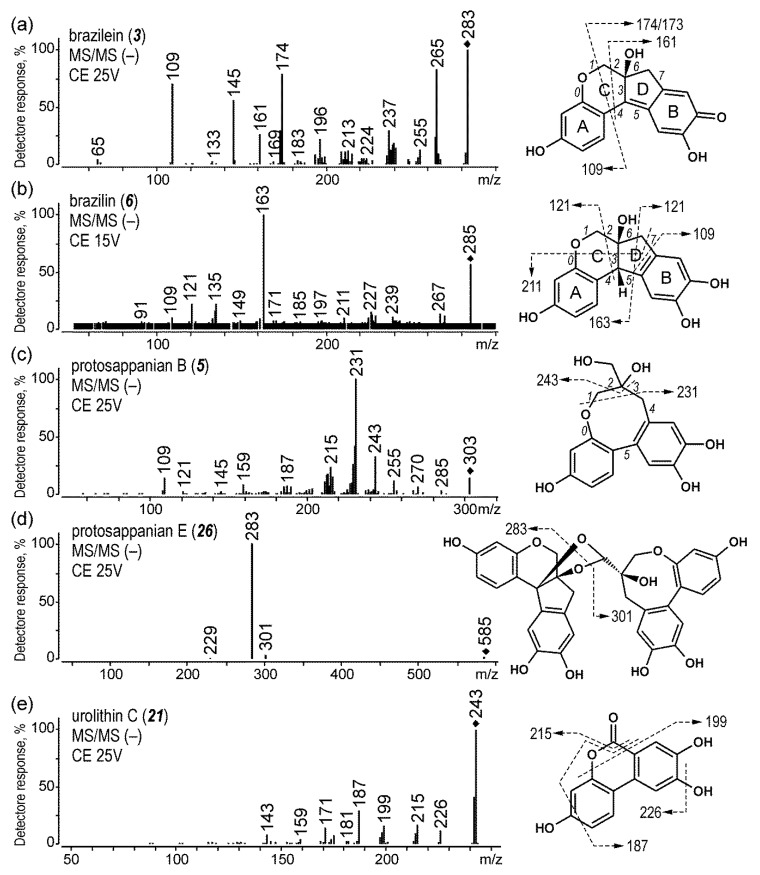
MS/MS spectra acquired in negative ion mode, and proposed fragmentation directions for (**a**) brazilein, (**b**) brazilin, (**c**) protosappanin B, (**d**) protosappanin E, and (**e**) urolithin C extracted from wool dyed with brazilwood.

The fragmentation of brazilin and brazilein proceeded according to two mechanisms, that is, the loss of small neutral molecules or the cleavage of internal rings (their MS/MS spectra and the proposed directions of their fragmentation are shown in Figure 3a,b). The MS/MS spectra of brazilein (***3***) (*m*/*z* 283 [M − H]^−^) showed signals at *m*/*z* 265 [M−H−H_2_O]^−^, 255 [M−H−CO]^−^, 237 [M−H−H_2_O−CO]^−^, and 196 [M−H−H_2_O−CO−C_2_HO]^−^. Moreover, C-ring fission led to the formation of intense ions at *m*/*z* 174 [^1,4^BD − H]^−^, 173 [^1,4^BD − H]^−^, 161 [^2,4^BD − H]^−^, 145 [^1,4^BD−H−CO]^−^, and 109 [^1,4^A − H]^−^. In the case of brazilin (***6***) (*m*/*z* 285 [M − H]^−^), fragmentation occurred mostly via D-ring cleavage, mainly giving the ion at *m*/*z* 163 [^5,6^A − H]^−^, as well as the lower signals at *m*/*z* 135 [^5,6^A−H−CO]^−^, 121 [^5,6^B − H]^−^ (or [^2,4^A − H]^−^), and 109 [^5,7^B − H]^−^. The intensities of other signals corresponding to the losses of small molecules (*m*/*z* 267 [M−H−H_2_O]^−^, 239 [M−H−CO_2_]^−^, 227 [M−H−H_2_O−CO]^−^, and 211 [M−H−C_3_H_6_O_2_]^−^) were rather low. On this basis, it can be assumed that compounds classified as brazilin-type homoisoflavonoids with a fused five-membered D-ring [43] decompose mostly via this D-ring, whereas their oxidized forms, with an extra unsaturated bond in a D-ring that prevent their fission, fragment via a heterocyclic C-ring.

The next two compounds eluted at 6.6 and 16.2 min were recognized to be protosappanin B (***5***) (*m*/*z* 303 [M − H]^−^) and prottosappanin E (***26***) (*m*/*z* 585 [M − H]^−^), respectively. The MS/MS spectra of the former one (Figure 3c) showed two main ions at *m*/*z* 243 [M−H−C_2_H_4_O_2_]^−^ and 231 [M−H−C_3_H_4_O_2_]^−^ formed by inner ring cleavage. The fragmentation of prottosappanin E, which is a combination of brazilin and protosappanin B molecules, was even simpler, since it resulted in only one signal at *m*/*z* 283 (Figure 3d). Probably, it was formed by the decomposition of bonds between both moieties.

Moreover, two brazilin-like compounds (***11***, ***28***) were also found (*t_R_* 8.4 and 16.4 min, both [M − H]^−^ at *m*/*z* 285). Their MS/MS spectra (Supplementary Materials, Figure S6) were like those of brazilin, but their structures remain unknown. Probably, they were brazilin isomers, or they belonged to homoisoflavans.

The compound eluted at 13.9 min was identified to be urolithin C (***21***) (*m*/*z* 243, [M − H]^−^, Figure 3e). Usually, it has been referred to as compound-type C, and its identity has been determined recently based on LC-ESI MS/MS, GC-MS, and NMR studies [36]. Since the structure of urolithin C is stabilized by resonance, the initiation of its fission required higher collision energies, and the fragmentation mostly led to the detachment of small molecules, such as OH (*m*/*z* 226), CO (*m*/*z* 215), CH_2_O (*m*/*z* 213), CO_2_ (*m*/*z* 199), and double CO (*m*/*z* 187).

#### 2.1.5. Logwood

Chromatograms acquired using spectrophotometric detection for the logwood extract showed several not-very-intense peaks at shorter retention times (Figure 1h). Two of them were identified to be hematein (***1***) and hematoxylin (***2***), a brazilin-type homoisoflavonoid [43]. Their MS/MS spectra (Supplementary Materials, Figure S7) showed that the decomposition of hematoxylin occurred via a D-ring, whereas hematein fragmented by the fission of a heterocyclic C-ring. Proposed fragmentation pathways are shown in Supplementary Materials, Figure S8.

The next four compounds observed in the chromatogram were found to be potential logwood markers (Supplementary Materials, Figure S9). The first two of them, coded as hc1 (***4***) and hc2 (***10***), were eluted at 5.8 min ([M − H]^−^ at *m*/*z* 319 → 259, 247, 241, 227) and 8.2 min ([M − H]^−^ at *m*/*z* 317 → 195, 167, 152, 125), respectively, but their structures have not been established. The other two compounds eluting at 7.2 min ([M − H]^−^ at *m*/*z* 603 → 301, 179, 137) and 9.4 min ([M − H]^−^ at *m*/*z* 581 → 281) were codded as hc3 (***7***) and hc4 (***15***), respectively. The higher *m*/*z* values of their quasi-molecular ions, low fragmentation, and generated product ions suggested these compounds could be a combined structure of two colorants. Therefore, since the product ions acquired for hc3 corresponded to the quasi-molecular and product ions of hematoxylin, hc3 (***7***) was considered to be a hematoxylin dimer.

#### 2.1.6. Sandalwood

Chromatograms acquired by a UV-Vis detector at 400 or 500 nm for the sandalwood extract showed four intense peaks (Figure 1i). According to the literature [44], it should contain santalins and santarubins, which was confirmed by MS/MS data. The first compound (t*_R_* 27.6 min), identified to be santalin A (***62***), gave the [M − H]^−^ ion at *m*/*z* 581 and its fragments at *m*/*z* 566, 551, and 523 (Figure 2f) corresponding to the single or double loss of CH_3_ radicals from methoxy groups and to the further detachment of CO from a carbonyl group, respectively.

Similar fragmentation pathways were observed for santalin B (***68***) (*t_R_* 29.8 min, [M − H]^−^ at *m*/*z* 595, Figure 2g) and santarubin A (***74***) (*t_R_* 30.9 min, [M − H]^−^ at *m*/*z* 609, Figure 2h). Their MS/MS spectra showed ions [M−H−CH_3_]^−^ at *m*/*z* 580 and 594, [M−H−2CH_3_]^−^ at *m*/*z* 565 and 579, [M−H−3CH_3_]^−^ at *m*/*z* 550 and 564, and [M−H−2CH_3_−CO]^−^ at *m*/*z* 537 and 551, respectively. Moreover, in the santalin B (***68***) spectra, there was also an intense signal at *m*/*z* 547 corresponding to the loss of H_2_O and two CH_3_ moieties.

The fragmentation of the last compound (t*_R_* 30.2 min, [M − H]^−^ at *m*/*z* 503, Supplementary Materials, Figure S10), which was coded as ps1 (***71***), proceeded according to the same pattern (*m*/*z* 488 [M−H−CH_3_]^−^, 473 [M−H−2CH_3_]^−^, and 445 [M−H−2CH_3_−CO]^−^), but the acquired MS/MS spectra turned out to be insufficient for determining the structure of the compound.

#### 2.1.7. Orchil

The chromatogram acquired in positive full scan mode by the ESI MS detector for the extract of wool dyed with orchil (*Roccella tinctoria* DC.) showed three significant as well as six minor peaks. According to the available literature data, these compounds corresponded to aminoorceins. To confirm the identity, the MS/MS spectra were acquired.

The most intense peak present in the chromatogram (Figure 1j) at 22.7 min was identified to be α-aminoorcein (***49***) ([M + H]^+^ at *m*/*z* 363). On the basis of the literature and by analogy with α-aminoorsein, it was assumed that the other two minor peaks corresponded to α-aminoorceinimine (***45***) ([M + H]^+^ at *m*/*z* 362) and α-hydroxyorcein (***58***) ([M + H]^+^ at *m*/*z* 364). Product ion spectra acquired by a triple-quadrupole mass spectrometer for the precursor ions of all three α-orceins indicated far-reaching similarity. Nonetheless, the identity of the MS/MS signals and determination of the fragmentation paths were very difficult, as most neutral losses could not be positively determined without high-resolution data due to the variety of possible isobaric fragments. For example, the loss of 17 Da could correspond to OH or NH_3_, whereas the loss of 29 Da could correlate with the detachment of CHO, C_2_H_5_, CH_2_N, or CH_2_NH. Since these losses are indistinguishable by triple quadrupole MS, the orchil extract was next examined using quadrupole-time-of-flight tandem mass spectrometry (QToF MS). High-resolution and high-accuracy product ions are presented in Table 2.

**Figure 4:**
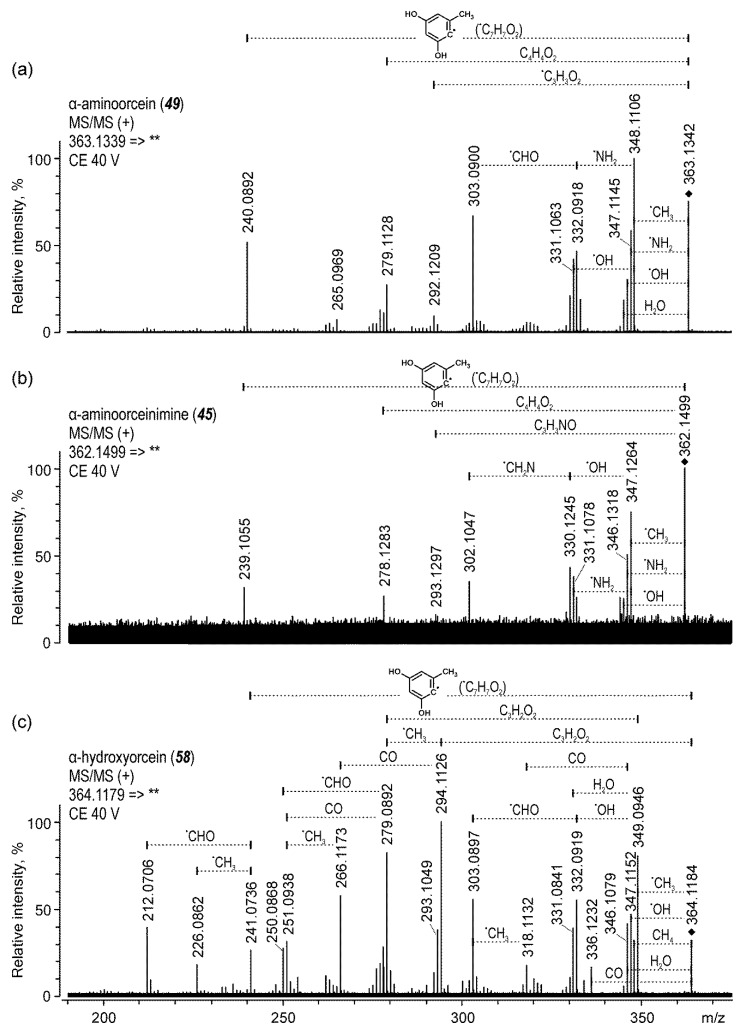
High resolution MS/MS spectra acquired in positive ion mode for α-aminoorcein (**a**), α-aminoorceinimine (**b**), and α-hydroxyorcein (**c**).

**Figure 5:**
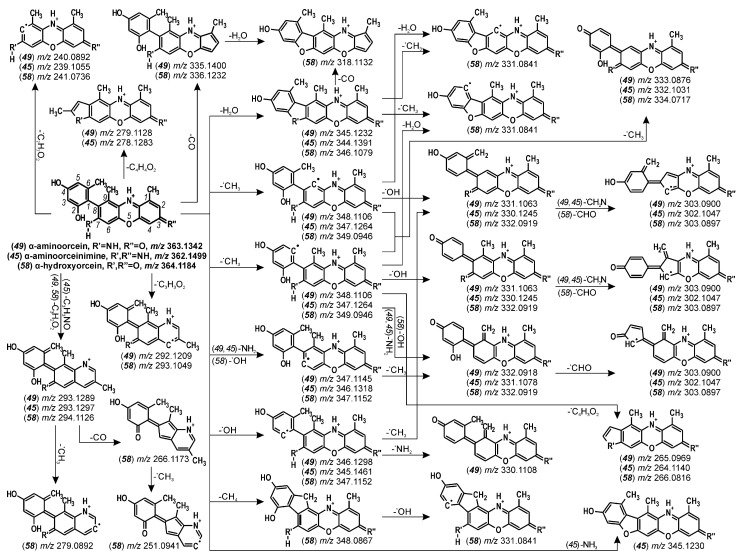
Proposed fragmentation pathways for α-aminoorcein (***49***), α-aminoorceinimine (***45***), and α-hydroxyorcein (***58***).

High-accuracy measurements of the [M + H]^+^ ions and the formulas calculated on their basis—that is, *m*/*z* 363.1342: C_21_H_19_N_2_O_4_; 362.1499: C_21_H_19_N_3_O_3_; and 364.1179: C_21_H_17_NO_5_—corresponded to α-aminoorcein (***49***), α-aminoorceinimine (***45***), and α-hydroxyorcein (***58***), respectively. Since these colorants are stabilized by their resonance structures, clear and legible MS/MS spectra were acquired only using higher collision energy (CE) values, such as 30–45 V (Figure 4), and the fragmentations started from the detachments of small radicals. Although thermodynamic arguments preclude the possibility of the loss of radicals from even-electron ions, the high-resolution mass spectra of orceins contradict this generally known theory. The most intense signals (at *m*/*z* 348.1106, 347.1264, and 349.0946 for α-aminoorcein, α-aminoorceinimine, and α-hydroxyorcein, respectively) corresponded to the loss of CH_3_. Except that, the [M + H−OH]^+^ ions were also present (*m*/*z* 346.1298, 345.1461, and 347.1152), but their origin was probably twofold. On the one hand, the hydroxyl radical could be detached from one of the two hydroxyl substituents of a phenyl ring (Figure 5). The same loss might also be achieved for α-hydroxyorcein by the homolytic cleavage of the hydroxyl group at the C-7 position of phenoxazin-3-one (*m*/*z* 347.1152). Since the other two compounds, α-aminoorcein and α-aminoorceinimine, are substituted at the C-7 position by an amino group, the detachment of the C-7 substituent led to the loss of an aminyl radical (NH_2_) and the formation of the *m*/*z* 347.1145 and 346.1318 ions, respectively. Even though radical loss from even-electron ions is rather unusual, this phenomenon has been already observed for prodiginines used as inks [45].

The fragmentation path also included the loss of small neutral fragments, such as H_2_O and CH_4_. It was observed mainly in the spectra of α-hydroxyorcein (*m*/*z* 348.0867) though, but the signals were less intense than those corresponding to the loss of radicals. The elimination of methane probably occurred between two methyl groups at the C-6 and C-9 positions, leading to the formation of the inner cyclopentadiene ring between the phenyl substituent and phenoxazin-3-one structure. A similar mechanism was responsible for the elimination of H_2_O from α-aminoorcein and α-hydroxyorcein (*m*/*z* 345.1232 and 346.1079, respectively) as well as of NH_3_ from α-aminoorceinimine (*m*/*z* 345.1230). These losses occurred between the C-2-hydroxyl group of the phenyl moiety and the C-7 substituent of the phenoxazine skeleton. Moreover, due to the presence of a carbonyl group at the C-3 position of α-aminoorcein and α-hydroxyorcein, one of the possible fragmentation paths also led to the detachment of the CO molecule (the *m*/*z* 335.1400 and 336.1232 ions, respectively), which was not observed for α-aminoorceinimine (Figure 5).

The next fragmentation stage was the further loss of the same small molecules (neutral and radical) from the primary product ions. The MS/MS spectra also showed the elimination of CHO or CH_2_N within the hydroxyl substituents or the amino group at the C-7 position of phenoxazine.

Apart from the loss of small neutrals or small radicals, the fragmentation of α-orceins also occurred with the detachment of larger fragments. One of them, the 2,4-dihydroxy-6-methylphenyl radical (C_7_H_7_O_2_), was created via a homolytic cleavage of the C-C bond between the phenyl ring and phenoxazine skeleton. The signals corresponding to this loss were observed in the spectra of α-aminoorcein, α-aminoorceinimine, and α-hydroxyorcein at *m*/*z* 240.0892, 239.1055, and 241.0736, respectively. Other losses were a result of a cross-ring fission. The primary loss of CH_3_ from the 2,4-dihydroxy-6-methylphenyl substituent probably triggered the ring fission and detachment of the C_4_H_3_O_2_ radical that was followed by the furan or pyrrole ring formation. Moreover, analogous ion structures were created by the elimination of the C_4_H_4_O_2_ molecules from the phenyl substituent, but these signals were present only in the MS/MS spectra of α-aminoorceinimine (*m*/*z* 278.1283) and α-aminoorcein (*m*/*z* 279.1128).

The next fragmentation path included the fission of the phenoxazine system and detachment of the structure between atoms 3 and 5. Since α-aminoorcein and α-hydroxyorcein are substituted at the C-3 position by a carbonyl group—and α-aminoorceinimine, by a primary ketimine group—this cleavage resulted in the loss of the C_3_H_2_O_2_ or C_3_H_3_NO molecules, respectively. In the case of α-hydroxyorcein, the [M + H−C_3_H_2_O_2_]^+^ ion (*m*/*z* 294.1126) was very intense, hence its further fragmentation and subsequent detachment of the CO molecule (*m*/*z* 266.1173) and CH_3_ radical (*m*/*z* 279.0892). Moreover, the alternative fission of the phenoxazine-3-one skeleton also led to the loss of the C_3_H_3_O_2_ fragment from quasi-molecular ions of α-aminoorcein and α-hydroxyorcein (giving the *m*/*z* 292.1209 and 293.1049 ions, respectively).

The peaks of three β- and three γ-orceins were also observed in the chromatogram, but since there were no significant differences between the MS/MS spectra of their β- and γ-isomers, distinction between their two forms was not possible. Moreover, peaks that corresponded to β- and γ-hydroxyorcein showed very low intensities.

**Figure 6:**
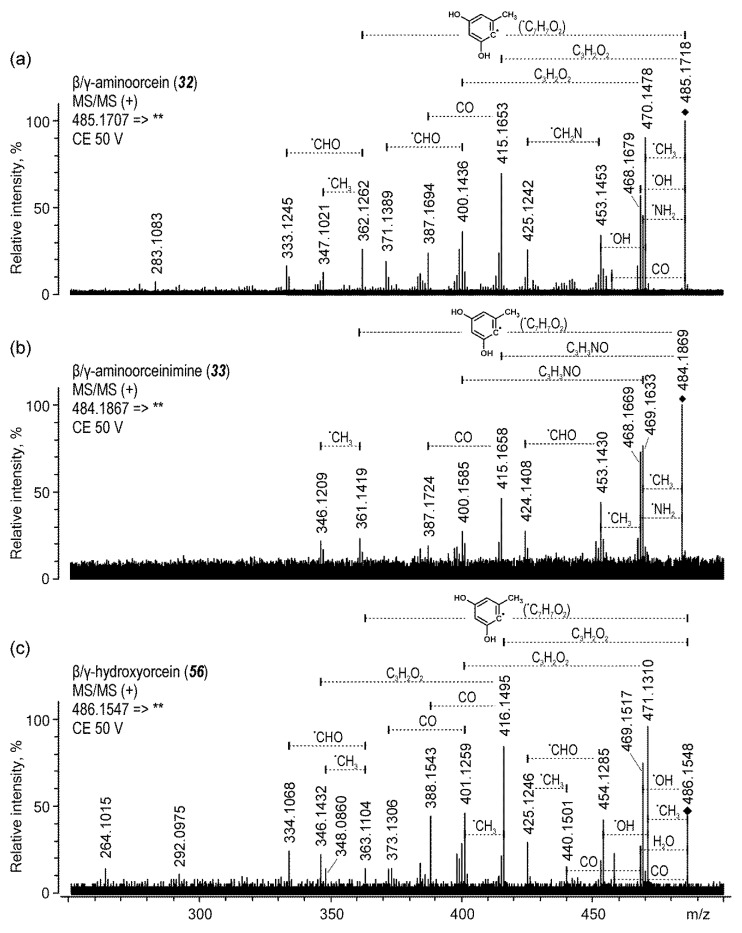
High-resolution MS/MS spectra acquired in negative ion mode for β/γ-aminoorcein (**a**), β/γ-aminoorceinimine (**b**), and β/γ-hydroxyorcein (**c**).

Since β- and γ-orceins are substituted by two 2,4-dihydroxy-6-methylphenyl groups (at the C-2 and C-8 positions), their fragmentation generated more product ions than the fragmentation of α-orceins. Nevertheless, these ions were produced, similarly as for α-orceins, according to the three paths: (1) the loss of small molecules, (2) phenyl ring fission, and (3) phenoxazine system fission. Most ions, however, especially those acquired for β- and γ-aminoorceins and hydroxyorceins (Figure 6), were formed as a result of various combination of these three pathways. MS/MS spectra showed signals corresponding to ions of mixed origin, such as [M + H−CH_3_−C_3_H_2_O_2_]^+^, [M + H−CH_3_−C_3_H_3_O_2_]^+^, [M + H−CO−C_3_H_2_O_2_]^+^, [M + H−CH_3_−C_7_H_7_O_2_]^+^, and [M + H−CHO−C_7_H_7_O_2_]^+^ (or [M + H−CO−C_3_H_3_NO]^+^ in the case of β- and γ-aminoorceinimine).

### 2.2. Protocol for Analyzing Historical Samples

**Figure 7:**
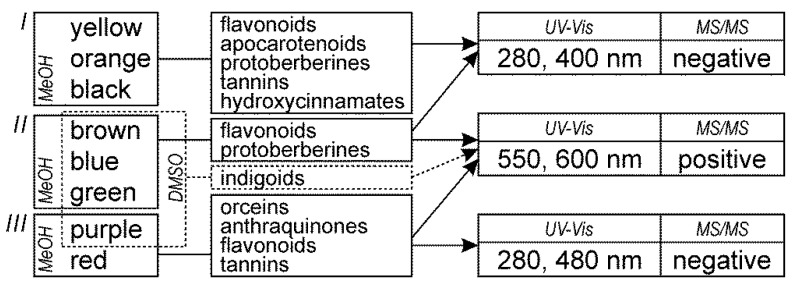
The integrated analytical protocol combining the extraction and analysis of colorants for the identification of natural dyes using HPLC-UV-Vis-ESI MS/MS.

The analytical protocol for the identification of natural dyes in historic textiles using HPLC coupled with UV-Vis and MS detections was proposed herein. This approach considered sample color (and thus also the extraction method), the UV-Vis and MS data acquired for the colorants (natural standards and markers in dyed fibers), and our expertise. Thus, according to the protocol (Figure 7), the detection of the colorants in the methanolic extracts from fibers should be conducted as follows: *(I)* yellow, orange, and black samples should be examined at 280 and 400 nm, in negative ion MS mode; *(II)* brown, blue, and green samples, at 280, 400, 550, and 600 nm, in both positive and negative ion MS modes; and *(III)* red and purple samples, at 280, 480, 550, and 600 nm, in both positive and negative ion MS modes; moreover, the DMSO extracts from (*IV*) brown, blue, green, and purple fibers should be analyzed at 550 and 600 nm, in positive ion MS mode. The protocol, recommended herein, was applied to identify the natural dyes used in historical samples, as described below.

### 2.3. Analysis of Historical Samples

The developed HPLC-UV-Vis-ESI MS/MS method was used to analyze 223 blue, purple, and red fibers taken from silk textiles dated from the 15th to 17th century and used in the vestments from the collections of twenty Krakow churches. The DMSO and methanol-water-formic acid extracts were analyzed using positive or positive and negative ion modes, respectively. The acquired results led to the identification of the natural dyes in the examined fibers, even though some of them were re-dyed with synthetic dyes. All the samples, identified colorants, and dyes are listed in Supplementary Materials, Table S1.

All the twenty-nine blue threads included in the set were dyed with indigo. Moreover, this dye was also identified in the next thirty-two samples of other colors (in fifteen at the trace level). Its use was proved by the presence of indigotin (***65***) in the extracts, always accompanied by isatin (***13***), a photodegradation product of indigotin. Indirubin was found only in some of these extracts. Since the composition of indigo colorants depends on the fermentation process for the indigo precursors, not on the origin of the plants used for this fermentation, indigo provenance could not be determined. Thus, the indigo could have been made from European or Asian plants from the genera *Indigofera*, *Isatis*, or others.

In 15th and 16th centuries, indigo was probably produced from woad (*Isatis tinctoria* L.), a native European plant that has been used on the Old Continent since antiquity. Although indigo from *Indigofer tinctoria* L. was imported to Europe after the discovery of the sea route to India, its use was much less likely at that time, since this dye was banned due to the allegedly “devilish origin” [46]. However, at the end of the 16th century, more and more Asian indigo came to Europe. Initially, it was used in combination with woad, but later on, during the 17th century, it replaced indigo from woad almost completely [44,46]. In consequence, threads taken from the younger textiles could be dyed using both *Indigofera* and *Isatis* species.

**Figure 8:**
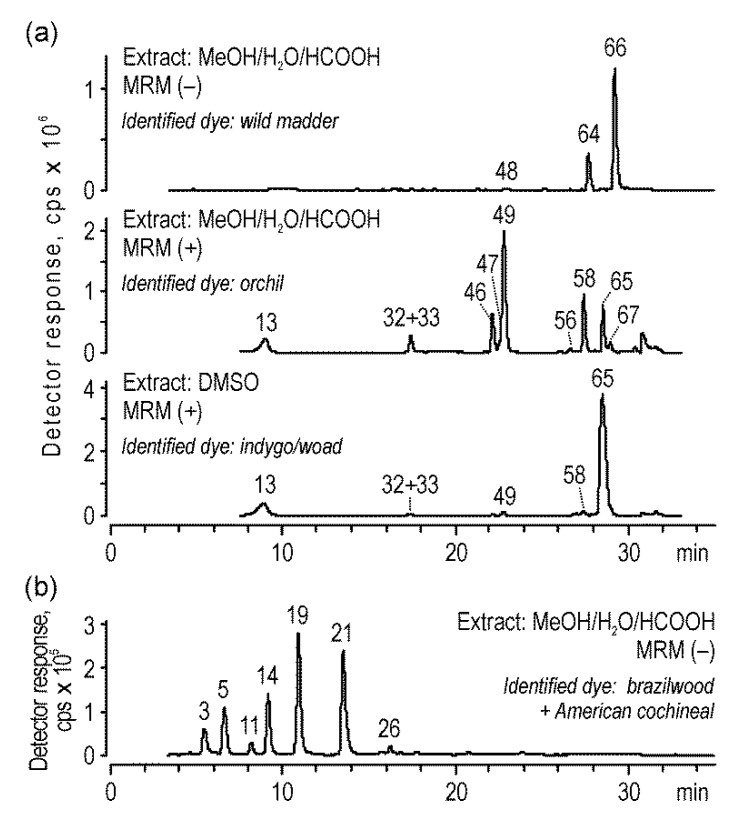
Chromatograms acquired for the extracts of the (**a**) blue sample (textile No. 282) and (**b**) red sample (textile No. 198) by the MS detector in negative and/or positive multiple reaction monitoring (MRM) modes; peak numbers are decoded in Table 1.

Although indigo was identified as a main dye in twenty-nine blue samples, it was used for individual dyeing in sixteen of them (in four of them, traces of other dyes were found as well). In another thirteen fibers, indigo was combined with other dyes and the fibers still remained blue. These dyes included American cochineal (one sample), weld (two samples), dyer′s broom (one sample), and, above all, orchil (ten samples, in two of them, together with wild madder (Figure 8a)).

Orchil, as a red-purple direct dye produced from lichenized fungi in the genus *Roccella*, has been known in Europe since ancient times, but knowledge about its use was lost with the fall of the Western Roman Empire. The dye returned as a textile dye at the end of the 13th century and the beginning of the 14th century. Although its use was restricted in France in the second half of the 17th century due to its poor light resistance, it was still used in other European countries [46]. In consequence, orchil was identified in thirty-three blue, purple, and red fibers taken from 15th- to 17th-century textiles; this dye was always used in a mixture with other dyes, never individually.

Seven out of eight purple samples were dyed either with a combination of orchil and indigo or with a ternary mixture of orchil, indigo, and American cochineal. In one purple sample, orchil was not present at all. A mixture of indigo and American cochineal was used to achieve the intended color instead.

Kermes was identified in seven red threads, always together with orchil. All those samples were taken from 15th- to 16th-century European textiles. Polish (*Porphyrophora polonica* L.) or Armenian cochineal (*Porphyrophora hamelii* L.) were found in the next six samples dated to the same period, but the unequivocal determination of the dye was impossible, since both scale insects belong to the same genus and their compositions are very similar to each other. However, the largest group of samples was dyed with American cochineal (most likely *Dactylopius coccus* Costa), which after arriving to Europe in 1523 [44] quickly displaced from the market other red dyes of animal origin such as kermes and Polish and Armenian cochineals.

American cochineal was identified by the presence of carminic acid (***19***) together with minor colorants, such as dcII (***18***), dcIV (***30***), dcVII (***36***), carminic acid derivative (***24***), kermesic acid (***52***), and flavokermesic acid (***51***). Although similar compounds were also found for the samples dyed with *Porphyrophora* species, they stood out with clearly higher contents of flavokermesic acid (***51***) and kermesic acid (***52***) as well as a complete absence of dcII (***18***) and trace presence of pp12 (***41***), pp14 (***53***), and pp15 (***60***) instead. In consequence, American cochineal was found as the only dye in one hundred and one thread samples, whereas in the next eleven threads, it was used together with annatto, brazilwood, dyer′s broom, and weld in single samples, as well as with indigo or its combination with orchil, as has already been mentioned. Nevertheless, most often, American cochineal was combined together with an unknown ellagitannin dye used as an organic mordant. These two dyes were identified together in eighteen samples.

The last identified animal-origin dye was lac dye (*Kerria lacca* Kerr or *K. chinensis* Mahdihassan). This Indian dye had been occasionally used in medieval Europe, but it gained popularity only in the second half of the 18th century [46]. Lac dye had been more widely known, however, in Muslim countries, including the Ottoman Empire, where it had been used to obtain crimson red. It was confirmed by the results, as lac dye was found in eight thread samples taken from 15th-century European textiles and in four fibers from 17th-century Turkish textiles.

The *Rubiaceae* dyes were identified in only nine samples (mostly dated to the 17th century); nevertheless, the different compositions of the anthraquinones in the extracts led to the distinguishing of three different chromatographic profiles. The first one, with high signals of alizarin (***55***), purpurin (***64***), and nordamnacanthal (***72***), corresponded to dyer′s madder (*Rubia tinctorum* L.). It was observed only in two extracts, which is understandable considering that, although madder as a European plant was widely cultivated on the continent, it was mainly used for dyeing wool, not silk. A similar chromatographic profile but without the peak of nordamnacanthal indicated the use of the *Galium* species, which was probably used in one sample. The high signal of rubiadin (***66***) together with the almost complete absence of alizarin corresponded to wild madder (*Rubia peregrina* L.). This dye was mostly used together with orchil and indigo to produce purple shades (Figure 8a).

Red color was obtained not only using anthraquinone dyes but also with flavonoid dyes. Thus, eleven red threads were dyed with brazilwood, which was used both individually as well as in combination with American cochineal (Figure 8b) or annatto (results published in [37,38]). Moreover, brazilwood was also found in nine samples of other colors (mostly yellow and orange). The origin of dyes, however, could not be determined precisely, since the same colorants were obtained from different tree species. One of them, sappanwood (*Caesalpinia sappan* L.), from southern Asia, was already known and used in medieval Europe, wherein the inner part of its trunks was used to produce brazilwood. In later centuries, the dye was also obtained from species imported from South America, such as *Caesalpinia echinata* L. and *Haematoxylum brasiletto* Karst. Furthermore, these trees were used for dyeing not only red but also yellow and orange.

## 3. Materials and Methods

Carminic acid, ellagic acid, indigotin, isatin, and purpurin were purchased from Fluka (Buchs, Switzerland); anthrarufin, anthraflavic acid, chrysazine, chrysophanol, hematein, quinizarin, and rubiadin were purchased from Sigma-Aldrich (St. Louis, MO, USA); and alizarin and hematoxylin were purchased from Riedel-de Haën (Seelze, Germany). All the chemicals (except alizarin) were of analytical chemical grade. Indirubin had been synthesized earlier by Puchalska [4], and chromatographic purity was estimated by HPLC/DAD at 280 nm. Kermesic acid was kindly donated by Dr. Ioannis Karapanagiotis (“Ormylia” Art Diagnosis Centre, Ormylia, Greece).

Alum AlK(SO_4_)_2_∙12H_2_O of analytical grade was from POCH (Gliwice, Poland). Sheep wool came from a rural farm in the Kuczbork commune of Northern Mazovia (Kuczbork, Poland). Methanol (LC/MS purity) and dimethyl sulfoxide (pure p.a.) were purchased from POCH (Gliwice, Poland); formic acid (LC/MS purity), from Fisher Scientific (Fair Lawn, NJ, USA); and hydrochloric acid (analytical grade, 35–38%), from AppliChem (Darmstadt, Germany). Demineralized water was made using a Milli-Q system Model Millipore Elix 3 (Molsheim, France).

Standard solutions of most colorants (0.2 mg∙mL^−1^) were prepared in methanol. Only indigotin and indirubin (0.1 mg∙mL^−1^) were dissolved in dimethyl sulfoxide (DMSO). Woolen yarns were mordanted, dyed, and extracted according to the procedure indicated previously [37]. Moreover, a 0.25 mg indigo sample was dissolved in 25 mL of DMSO. The solutions were kept in an ultrasonic bath for 5 min, and the obtained solutions was diluted 10 times with methanol.

The 223 silk fibers (red, purple, and blue) were taken from silk textiles that were dated to the 15th to 17th century and used in the vestments belonging to the collections of twenty Krakow churches (all the samples are listed in Supplementary Material, Table S1).

The purple and blue fibers were extracted twice, using two extraction methods consecutively, the first one with dimethylsulfoxide (DMSO), and the second one with acidic-methanol extractant. The red fibers were extracted only with the second procedure. The extraction procedures have been described in detail by Lech [37]. 

Chromatographic analyses were performed using a 1220 Infinity II LC System (Agilent Technologies, Waldbronn, Germany) with a Zorbax SB-Phenyl column (4.6 × 150 mm, 3.5 μm, 80 Å, Agilent Technologies); a Zorbax SB-Phenyl precolumn (4.6 × 12.5 mm, 5.0 µm, Agilent Technologies); and a mixture of methanol, water, and formic acid as a mobile phase. Detection was carried out with a 1220 Compact Variable Wavelength Detector and a 1200 Variable Wavelength Detector (Agilent Technologies, Waldbronn, Germany), as well as with a 6460 Triple Quad LC/MS system with *JetStream* Technology (Agilent Technologies, Waldbronn, Germany). The full scan chromatograms and spectra were acquired for *m*/*z* 100–1000. Quasi-molecular ions of the colorants were fragmented using 15, 25, 35, and 45 V of collision energy (CE). The MS/MS spectra were acquired from *m*/*z* 50 to the *m*/*z*-value of the precursor ion + 20 to achieve an upper limit of around 20 *m*/*z* above the *m*/*z* of each fragmented ion. The parameters of the method were described in detail by Lech [37]. The final method was developed in dynamic multiple reaction monitoring (dMRM) mode given the most intense precursor and product ion pairs (transitions) of the identified dye markers. The optimal collision energy for each transition was selected manually. Detailed settings (the retention times, fragmentor values for each precursor ion, MRM transitions, and CEs of the new colorants) are provided in Table 1.

High-accuracy mass spectra of the natural orceins (phenoxazines) were acquired using a UHD Accurate-Mass 6540 Q-TOF LC/MS system with *JetStream* Technology (Agilent Technologies, USA). The MS was operated in positive ionization MS/MS mode. The following parameters were applied: probe voltage, 3500 V; nozzle voltage, 1000 V; gas temperature, 300 °C; gas flow, 8 L·min^−1^; nebulizer pressure, 35 psig; sheath gas temperature, 350 °C; sheath gas flow, 11 L·min^−1^; fragmentor, 120 V; collision energies, 30, 40, and 50 V; reference masses, *m*/*z* 121.05087300 and 922.00979800.

The analyses were performed using the MassHunter Workstation software (Agilent Technologies, USA).

## 4. Conclusions

A phenyl HPLC column and UV-Vis-ESI MS/MS technique were used to separate and characterize colorants (flavonoids, homoisoflavonoids, anthraquinones, indigoids, and orceins) present in the extracts of ten natural dyes (American cochineal, brazilwood, indigo, kermes, lac dye, logwood, madder, orchil, sandalwood, and Polish cochineal). Tandem mass spectrometric detection provided information on the structures of unknown colorants eluted from the HPLC column. Several colorants were identified in this way, including protosappanin B, protosappanin E, santalin A, santalin B, santarubin A, nordamnacanthal, lucidin, erythrolaccin, and deoxyerythrolaccin, but the structures of some compounds (from brazilwood, logwood, madder, and sandalwood) are still pending. Moreover, complex fragmentation pathways of α-, β- and γ- aminoorceins, hydroxyorceins, and aminoorceinimines extracted from orchil-dyed wool have been defined the first time according to our knowledge on the basis of high-resolution mass spectrometry data acquired by QToF MS. The results have shown that the fragmentation is twofold. It occurs by the loss of small neutrals and radicals, as well as by the loss or fission of the aromatic rings.

MS/MS data have been used not only to identify new colorants but also to expand the existing dMRM method with 60 new dye markers. It has resulted in the development, as far as we know, of the first universal and comprehensive approach, that includes 176 colorants, intended for the identification of natural dyes in historical objects. Furthermore, a general analytical protocol has been developed for the identification of the natural dyes used in historical objects, antiques, and works of art. It involves both extraction and analysis steps (including UV-Vis detection wavelengths and MS ionization modes) and also considers fiber colors and the physicochemical properties of presumed dyes. This approach has been used to analyze 223 red, purple, and blue fibers taken from the silk textiles used in the vestments belonging to the collections of twenty Krakow churches. It has led to the identification of several dyes, such as orchil, brazilwood, madder, wild madder, indigo, lac dye, kermes, and different species of cochineals. The results of this study have completed the picture of natural dyes used in the most valuable textiles of European and Near Eastern origin dated to the 15th to 17th century.

## Figures and Tables

**Table 1:** High-performance liquid chromatography coupled with spectrophotometric detection and tandem mass spectrometric detection with electrospray ionization (HPLC-UV-Vis-ESI MS/MS) characterization of color compounds.

No	Compound Name	*t_R_*, min	[M − H]^−^, *m*/*z*	Frag., V	Product Ions, *m*/*z* (CE, V)	λ_max_, nm
1	hematein *	4.0	299	130	281 (8), 253 (15), 174 (20), 125 (20)	284, 383
2	hematoxylin *	5.2	301	150	283 (17), 179 (15), 137 (21), 123 (29)	279, 397
3	brazilein	5.5	283	130	265 (25), 174 (25), 145 (25), 109 (25)	
4	hc1	5.8	319	90	259 (15), 247 (15), 241 (15), 227 (15)	
5	protosappanin B	6.6	303	130	231 (15)	
6	brazilin	7.1	285	110	163 (25), 135 (30), 121 (30)	
7	hc3 (hematoxylin dimer)	7.2	603	150	301 (20), 179 (20)	
8	laccaic acid E	7.9	494	90	450 (10), 406 (20)	228, 288, 492
9	deoxyerythrolaccin di-*O*-hexoside (pp2)	8.0	593	170	431 (25), 269 (35)	
10	hc2	8.2	317	90	195 (15), 152 (15), 125 (30)	
11	ceas1 (brazilin-like)	8.4	285	130	257 (15), 243 (25), 214 (25)	
12	kermesic acid di-*C*-hexoside (pp3)	8.5	653	170	609 (20), 357 (30), 327 (30)	
14	caesD	9.3	303	130	245 (10), 227 (10), 217 (25)	
15	hc4 (dimer)	9.4	581	150	281 (25)	
16	laccaic acid C	9.5	538	90	494 (10), 450 (20)	228, 288, 492
17	flavokermesic acid *O*-hexoside (pp6/ppI)	10.3	475	170	431 (15), 269 (25)	285, 339, 399
18	flavokermesic acid 2-*C*-glucoside (dcII)	10.5	475	170	431 (13), 341 (22), 311 (22), 282 (41)	287, 435
19	carminic acid *	11.2	491	170	447 (14), 357 (22), 327 (22), 299 (34)	276, 310, 496
20	kermesic acid *O*-hexoside (pp7/ppII)	11.8	491	170	447 (15), 285 (25)	276, 466
21	urolithin C	13.9	243	130	215 (25), 199 (25), 187 (25)	
22	dc3	14.5	535	170	473 (20), 445 (30), 415 (25)	
23	flavokermesic acid 6-*O*-glucoside (dc*O*fka)	14.8	475	170	431 (20), 268 (35), 240 (45)	280, 342, 431
24	carminic acid derivative	15.1	519	170	475 (15), 357 (25), 327 (25), 298 (40)	
25	dc4	15.5	519	170	397 (30), 385 (25), 327 (35)	
26	protosappanin E	16.2	585	90	283 (15)	
27	kermesic acid *O*-hexoside (pp9)	16.3	491	170	447 (18), 284 (25)	
28	caes2 (brazilin-like)	16.4	285	130	161 (15), 134 (25)	
29	deoxyerythrolaccin *O*-hexoside (pp10)	16.5	431	170	269 (25)	
30	kermesic acid 7-*C*-glucofuranoside (dcIV)	16.8	491	170	447 (15), 357 (25), 327 (35), 299 (40)	277, 314, 493
34	xantholaccaic acid B	17.4	479	90	435 (10), 391 (25)	
35	lucidin *O*-primeveroside	17.9	563	130	269 (10), 251 (45)	246, 266, 342, 407
36	kermesic acid 7-*C*-glucofuranoside (dcVII)	18.4	491	170	447 (15), 357 (25), 327 (30), 299 (35)	277, 312, 492
38	ruberthyric acid	18.5	533	130	239 (18)	228, 258, 334, 416
39	laccaic acid B	19.2	495	90	451 (10), 407 (20), 389 (35)	230, 288, 492
40	xantholaccaic acid A	19.4	520	90	476 (10), 432 (25)	230, 288, 492
41	deoxyerythrolaccin *O*-hexoside (pp12)	21.5	431	170	268 (30)	
42	anthraflavic acid *	21.6	239	130	211 (26), 210 (30), 195 (22), 182 (42)	240, 273, 299, 346
43	laccaic acid A	21.6	536	90	492 (10), 448 (18), 430 (30), 358 (45)	228, 288, 492
48	rubiadin *O*-primeveroside	22.6	547	130	253 (20)	
50	anthragallol	23.6	255	130	227 (25), 153 (35), 125 (35)	
51	flavokermesic acid	23.6	313	90	269 (10)	284, 342, 431
52	kermesic acid *	24.2	329	90	285 (10)	274, 308, 492
53	kermesic acid *O*-hexoside derivative (1) (pp14)	25.5	589	170	545 (20), 357 (25), 327 (25)	
54	lucidin	25.7	299	130	251 (15), 223 (30), 195 (35)	
55	alizarin *	26.4	239	170	211 (26), 210 (30)	248, 274, 324, 429
57	deoxyerythrolaccin	27.2	269	130	241 (25), 225 (25)	
59	anthrarufin *	27.4	239	170	211 (26), 182 (45)	225, 252, 285, 417
60	kermesic acid *O*-hexoside derivative (2) (pp15)	27.4	617	170	545 (20), 357 (25), 327 (25)	
61	xanthopurpurin	27.5	239	130	211 (25), 195 (25)	
62	santalin A	27.6	581	130	566 (25), 551 (32), 523 (40)	
63	erythrolaccin	27.9	285	130	257 (25), 241 (25)	
64	purpurin *	28.3	255	130	227 (22), 171 (30), 129 (38), 101 (45)	255, 290, 482
66	rubiadin *	29.0	253	110	225 (25), 209 (22), 195 (55)	245, 278, 330, 411
68	santalin B	29.8	595	130	580 (25), 565 (35)	
69	chrysazin *	29.8	239	170	211 (26)	223, 252, 283, 428
70	quinizarin *	30.1	239	210	211 (18)	224,248, 278, 324, 479
71	ps1(santalin-like)	30.2	503	130	488 (20), 473 (25), 445 (25)	
72	nordamnacanthal	30.5	267	90	239 (14), 211 (25), 195 (34)	259, 294, 418
73	chrysophanol *	30.8	253	170	225 (26)	225, 256, 277, 287, 429
74	santarubin A	30.9	609	130	594 (25), 579 (25), 551 (40)	
75	atranorin *	31.0	373	90	177 (10), 163 (14), 133 (22)	
76	rt1 (alizarin-licidin *O*-dimer)	32.1	491	150	251 (25), 239 (35)	
13	isatin *	8.8	148	90	130 (15), 102 (25), 92 (20), 77 (25), 65 (30)	296, 413
31	indigoid compound A	17.2	262	90	235 (30), 219 (30), 190 (40), 120 (30)	
32	β/γ-aminoorcein	17.2	485	140	470 (45), 415 (40), 362 (42)	
33	β/γ-aminoorceinimine	17.3	484	140	469 (50), 468 (42), 361 (50)	
37	indigoid compound B	18.4	262	90	234 (20), 219 (20), 31 (30)	
44	β/γ-hydroxyorcein	21.7	486	140	471 (45), 469 (40), 416 (40)	
45	α-aminoorceinimine	21.9	362	140	347 (38), 331 (45), 278 (40)	
46	β/γ-aminoorceinimine	22.0	484	140	469 (45), 424 (55), 362 (42)	
47	β/γ-aminoorcein	22.3	485	140	470 (42), 415 (40), 362 (42)	
49	α-aminoorcein	22.7	363	140	348 (28), 347 (32), 303 (40), 240 (36)	
56	β/γ-hydroxyorcein	26.6	486	140	471 (45), 416 (42)	
58	α-hydroxyorcein	27.3	364	140	349 (30), 344 (25), 294 (25), 279 (40)	
65	indigotin *	28.7	263	90	235 (23), 219 (19), 206 (39), 132 (35), 77 (50)	291, 620 *^#^*
67	indirubin *	29.2	263	170	235 (19), 219 (23), 190 (43)	257, 550 *^#^*

* Data determined for standard solutions and presented in [37], *^#^* absorption maxima determined for DMSO solutions.

**Table 2:** Product ions acquired using high-resolution MS/MS for protonated ions of orceins extracted from wool dyed with orchil.

Compound	*t_R_*, min	[M + H]^+^, *m*/*z*	Fragment ion, *m*/*z*	Calc. *m*/*z*	Formula	Diff, ppm	Abund %
β/γ-aminoorcein (***32***)	17.2	485.1718		485.1707	C_28_H_25_N_2_O_6_	−2.20	100.0
			470.1478	470.1472	C_27_H_22_N_2_O_6_	−1.24	90.3
			469.1526	469.1520	C_28_H_23_NO_6_	−1.36	45.4
			468.1679	468.1680	C_28_H_24_N_2_O_5_	0.21	57.0
			467.1615	467.1602	C_28_H_23_N_2_O_5_	−2.91	16.4
			457.1760	457.1758	C_27_H_25_N_2_O_5_	−0.51	14.1
			453.1453	453.1445	C_27_H_21_N_2_O_5_	−1.86	34.2
			425.1242	425.1258	C_26_H_19_NO_5_	3.79	25.7
			415.1653	415.1652	C_25_H_23_N_2_O_4_	−0.18	69.5
			414.1571	414.1574	C_25_H_22_N_2_O_4_	0.82	23.8
			400.1436	400.1418	C_24_H_20_N_2_O_4_	−4.52	36.3
			399.1367	399.1339	C_24_H_19_N_2_O_4_	−6.89	26.1
			387.1694	387.1703	C_24_H_23_N_2_O_3_	2.26	23.9
			371.1389	371.1390	C_23_H_19_N_2_O_3_	0.22	18.8
			362.1262	362.1261	C_21_H_18_N_2_O_4_	−0.43	26.2
			347.1383	347.1390	C_21_H_19_N_2_O_3_	2.17	10.9
			347.1021	347.1026	C_20_H_15_N_2_O_4_	1.45	12.9
			334.1303	334.1312	C_20_H_18_N_2_O_3_	2.60	10.2
			333.1245	333.1234	C_20_H_17_N_2_O_3_	−3.35	16.6
			283.1083	283.1077	C_16_H_15_N_2_O_3_	−2.09	7.5
β/γ-aminoorceinimine (***33***)	17.3	484.1869		484.1867	C_28_H_26_N_3_O_5_	−0.40	100.0
			469.1633	469.1632	C_27_H_23_N_3_O_5_	−0.20	76.6
			468.1669	468.1680	C_28_H_24_N_2_O_5_	2.25	73.1
			467.1836	467.1840	C_28_H_25_N_3_O_4_	0.78	23.4
			467.1615	467.1602	C_28_H_23_N_2_O_5_	−2.97	22.5
			466.1789	466.1761	C_28_H_24_N_3_O_4_	−5.95	14.0
			453.1430	453.1445	C_27_H_21_N_2_O_5_	3.27	43.9
			424.1408	424.1418	C_26_H_20_N_2_O_4_	2.32	27.5
			415.1658	415.1652	C_25_H_23_N_2_O_4_	−1.28	46.4
			414.1585	414.1574	C_25_H_22_N_2_O_4_	−2.63	21.0
			400.1685	400.1656	C_24_H_22_N_3_O_3_	−7.33	27.4
			387.1724	387.1703	C_24_H_23_N_2_O_3_	−5.30	19.1
			361.1419	361.1421	C_21_H_19_N_3_O_3_	0.63	23.2
			346.1209	346.1186	C_20_H_16_N_3_O_3_	−6.65	21.9
β/γ-hydroxyorcein (***44***)	21.7	486.1561		486.1547	C_28_H_24_NO_7_	−2.86	54.2
			471.1309	471.1313	C_27_H_21_NO_7_	0.87	100.0
			470.1231	470.1234	C_27_H_20_NO_7_	0.57	16.9
			469.1515	469.1520	C_28_H_23_NO_6_	1.13	59.1
			468.1450	468.1442	C_28_H_22_NO_6_	−1.69	20.8
			458.1590	458.1598	C_27_H_24_NO_6_	1.77	16.1
			454.1285	454.1285	C_27_H_20_NO_6_	−0.07	35.6
			440.1488	440.1493	C_27_H_22_NO_5_	1.05	13.6
			425.1266	425.1258	C_26_H_19_NO_5_	−1.81	37.2
			416.1491	416.1493	C_25_H_22_NO_5_	0.58	63.6
			415.1421	415.1414	C_25_H_21_NO_5_	−1.66	30.6
			401.1258	401.1258	C_24_H_19_NO_5_	−0.10	37.4
			400.1185	400.1179	C_24_H_18_NO_5_	−1.37	23.3
			388.1544	388.1543	C_24_H_22_NO_4_	−0.15	40.2
			386.1010	386.1023	C_23_H_16_NO_5_	3.37	12.2
			384.1234	384.1204	C_21_H_20_O_7_	−7.71	12.5
			373.1311	373.1309	C_23_H_19_NO_4_	−0.40	21.9
			372.1236	372.1230	C_23_H_18_NO_4_	−1.64	12.8
			363.1113	363.1101	C_21_H_17_NO_5_	−3.28	33.5
			348.0864	348.0866	C_20_H_14_NO_5_	0.57	19.3
			346.1429	346.1438	C_22_H_20_NO_3_	2.57	15.8
			334.1078	334.1074	C_20_H_16_NO_4_	−1.11	25.9
			293.1053	293.1046	C_18_H_15_NO_3_	−2.46	17.3
			264.1032	264.1019	C_17_H_14_NO_2_	−4.96	14.4
α-aminoorceinimine (***45***)	21.9	362.1499		362.1499	C_21_H_20_N_3_O_3_	−0.08	100.0
			347.1264	347.1264	C_20_H_17_N_3_O_3_	0.04	73.8
			346.1318	346.1312	C_21_H_18_N_2_O_3_	−1.85	47.4
			345.1461	345.1472	C_21_H_19_N_3_O_2_	4.01	19.2
			345.1230	345.1234	C_21_H_17_N_2_O_3_	1.19	10.3
			344.1391	344.1394	C_21_H_18_N_3_O_2_	0.81	17.6
			332.1031	332.1030	C_19_H_14_N_3_O_3_	−0.42	19.7
			331.1078	331.1077	C_20_H_15_N_2_O_3_	−0.27	33.1
			330.1245	330.1237	C_20_H_16_N_3_O_2_	−2.39	38.3
			302.1047	302.1050	C_19_H_14_N_2_O_2_	0.93	26.3
			293.1297	293.1284	C_18_H_17_N_2_O_2_	−4.26	17.5
			278.1283	278.1288	C_17_H_16_N_3_O	1.62	24.0
			264.1140	264.1131	C_16_H_14_N_3_O	−3.41	12.0
			262.1099	262.1101	C_17_H_14_N_2_O	0.76	9.3
			239.1055	239.1053	C_14_H_13_N_3_O	−0.80	30.4
β/γ-aminoorceinimine (***46***)	22.0	484.1868		484.1867	C_28_H_26_N_3_O_5_	−0.28	100.0
			469.1629	469.1632	C_27_H_23_N_3_O_5_	0.60	61.5
			468.1668	468.1680	C_28_H_24_N_2_O_5_	2.58	71.4
			467.1783	467.1840	C_28_H_25_N_3_O_4_	12.18	17.6
			466.1775	466.1761	C_28_H_24_N_3_O_4_	−3.05	11.1
			453.1436	453.1445	C_27_H_21_N_2_O_5_	1.94	26.5
			452.1613	452.1605	C_27_H_22_N_3_O_4_	−1.70	25.1
			424.1422	424.1418	C_26_H_20_N_2_O_4_	−0.94	17.8
			415.1630	415.1652	C_25_H_23_N_2_O_4_	5.30	16.6
			400.1664	400.1656	C_24_H_22_N_3_O_3_	−2.05	12.5
			361.1429	361.1421	C_21_H_19_N_3_O_3_	−2.10	24.1
			346.1196	346.1186	C_20_H_16_N_3_O_3_	−2.83	8.2
β/γ-aminoorcein (***47***)	22.3	485.1721		485.1707	C_28_H_25_N_2_O_6_	−2.94	100.0
			470.1473	470.1472	C_27_H_22_N_2_O_6_	−0.30	86.1
			469.1511	469.1520	C_28_H_23_NO_6_	1.83	35.2
			468.1681	468.1880	C_28_H_24_N_2_O_5_	−0.21	53.5
			467.1602	467.1602	C_28_H_23_N_2_O_5_	−0.06	14.5
			457.1762	457.1758	C_27_H_25_N_2_O_5_	−0.87	15.7
			453.1442	453.1445	C_27_H_21_N_2_O_5_	0.73	29.4
			425.1266	425.1258	C_26_H_19_NO_5_	−1.79	20.7
			415.1657	415.1652	C_25_H_23_N_2_O_4_	−1.28	63.2
			414.1575	414.1574	C_25_H_22_N_2_O_4_	−0.17	21.0
			400.1412	400.1418	C_24_H_20_N_2_O_4_	1.50	34.6
			399.1371	399.1339	C_24_H_19_N_2_O_4_	−7.97	22.0
			387.1697	387.1703	C_24_H_23_N_2_O_3_	1.58	28.0
			371.1387	371.1390	C_23_H_19_N_2_O_3_	0.86	14.9
			362.1262	362.1261	C_21_H_18_N_2_O_4_	−0.30	22.5
			347.1375	347.1390	C_21_H_19_N_2_O_3_	4.26	7.6
			347.1022	347.1026	C_20_H_15_N_2_O_4_	1.24	19.5
			334.1302	334.1312	C_20_H_18_N_2_O_3_	2.90	9.0
			333.1238	333.1234	C_20_H_17_N_2_O_3_	−1.17	12.0
			283.1068	283.1077	C_16_H_15_N_2_O_3_	3.21	4.4
α-aminoorcein (***49***)	22.7	363.1342		363.1339	C_21_H_19_N_2_O_4_	−0.73	75.2
			348.1106	348.1105	C_20_H_16_N_2_O_4_	−0.41	100.0
			347.1145	347.1152	C_21_H_19_N_2_O_4_	2.06	58.7
			346.1298	346.1312	C_21_H_18_N_2_O_3_	3.93	30.1
			346.1063	346.1074	C_21_H_16_NO_4_	3.01	11.0
			345.1232	345.1234	C_21_H_17_N_2_O_3_	0.41	18.8
			335.1400	335.1400	C_20_H_19_N_2_O_3_	−2.78	2.3
			333.0876	333.0870	C_19_H_13_N_2_O_4_	−1.81	19.1
			332.0918	332.0917	C_20_H_14_NO_4_	−0.21	46.7
			331.1063	331.1077	C_20_H_15_N_2_O_3_	4.14	42.1
			330.1108	330.1125	C_21_H_16_NO_3_	5.03	21.2
			303.0900	303.0890	C_19_H_13_NO_3_	−3.35	67.2
			293.1289	293.1285	C_18_H_17_N_2_O_2_	−1.39	4.8
			292.1209	292.1206	C_18_H_16_N_2_O_2_	−0.83	9.5
			279.1128	279.1128	C_17_H_15_N_2_O_2_	0.03	27.5
			265.0969	265.0969	C_16_H_13_N_2_O_2_	0.80	7.4
			240.0892	240.0893	C_14_H_12_N_2_O_2_	0.43	51.9
β/γ-hydroxyorcein (***56***)	26.6	486.1548		486.1547	C_28_H_24_NO_7_	−0.31	40.7
			471.1310	471.1313	C_27_H_21_NO_7_	0.63	100.0
			469.1517	469.1520	C_28_H_23_NO_6_	0.52	74.3
			468.1438	468.1442	C_28_H_22_NO_6_	0.82	23.3
			458.1594	458.1598	C_27_H_24_NO_6_	0.90	22.6
			454.1285	454.1285	C_27_H_20_NO_6_	0.09	41.9
			440.1501	440.1493	C_27_H_22_NO_5_	−2.00	12.9
			425.1246	425.1258	C_26_H_19_NO_5_	2.77	28.8
			416.1487	416.1493	C_25_H_22_NO_5_	1.31	83.8
			401.1259	401.1258	C_24_H_19_NO_5_	−0.22	45.8
			400.1187	400.1179	C_24_H_18_NO_5_	−1.91	28.1
			388.1543	388.1543	C_24_H_22_NO_4_	−0.02	43.8
			384.1189	384.1204	C_21_H_20_O_7_	3.72	17.1
			373.1306	373.1309	C_23_H_19_NO_4_	0.75	13.7
			372.1236	372.1230	C_23_H_18_NO_4_	−1.45	13.5
			363.1104	363.1101	C_21_H_17_NO_5_	−0.87	13.8
			348.0860	348.0866	C_20_H_14_NO_5_	1.95	14.0
			346.1432	346.1438	C_22_H_20_NO_3_	1.67	22.0
			334.1068	334.1074	C_20_H_16_NO_4_	1.79	24.1
			292.0975	292.0968	C_18_H_14_NO_3_	−2.17	9.5
			264.1015	264.1019	C_17_H_14_NO_2_	1.72	13.9
α-hydroxyorcein (***58***)	27.3	364.1184		364.1179	C_21_H_18_NO_5_	−1.17	30.9
			349.0946	349.0945	C_20_H_15_NO_5_	−0.45	79.6
			348.0867	348.0866	C_20_H_14_NO_5_	−0.10	29.5
			347.1152	347.1152	C_21_H_17_NO_4_	−0.07	45.6
			346.1079	346.1074	C_21_H_16_NO_4_	−1.60	40.5
			336.1232	336.1230	C_20_H_18_NO_4_	−0.49	15.1
			334.0717	334.0710	C_19_H_12_NO_5_	−2.12	8.8
			332.0919	332.0917	C_20_H_14_NO_4_	−0.43	55.3
			331.0841	331.0839	C_20_H_13_NO_4_	−0.49	38.9
			318.1132	318.1125	C_20_H_16_NO_3_	−2.32	16.2
			303.0897	303.0890	C_19_H_13_NO_3_	−2.22	54.6
			294.1126	294.1125	C_18_H_16_NO_3_	−0.34	100.0
			293.1049	293.1046	C_18_H_15_NO_3_	−0.95	37.8
			279.0892	279.0890	C_17_H_13_NO_3_	−0.57	81.4
			266.1173	266.1176	C_17_H_16_NO_2_	0.98	57.6
			266.0816	266.0816	C_16_H_12_NO_3_	−1.69	8.0
			251.0938	251.0941	C_16_H_13_NO_2_	1.22	30.5
			250.0868	250.0863	C_16_H_12_NO_2_	−2.33	25.9
			241.0736	241.0733	C_14_H_11_NO_3_	−0.96	25.9
			226.0862	226.0863	C_14_H_12_NO_2_	0.21	16.4
			212.0706	212.0706	C_13_H_10_NO_2_	0.27	38.9

## Supplementary Materials

Figure S1: Indigo: MS/MS spectra of indigotin, indirubin, indigo compound A, and indigo compound A acquired in positive ion mode. Figure S2: Chemical structures of laccaic acids. Figure S3: Lac dye: MS/MS spectra of laccaic acid E, laccaic acid C, xantholaccaic acid B, laccaic acid B, xantholaccaic acid A, and laccaic acid A, acquired in negative ion mode. Figure S4: Madder: MS/MS spectra of rt1 (alizarin–lucidin dimer). Figure S5: Madder: MS/MS spectra of rubiadin *O*-primeveroside, ruberthyric acid, and lucidin *O*-primeveroside acquired in negative ion mode. Figure S6: Brazilwood: MS/MS spectra of brazilin-like compounds in negative ion mode. Figure S7: Logwood: MS/MS spectra of hematoxylin and hematein acquired in negative ion mode. Figure S8: Proposed fragmentation pathways for a) hematoxylin and b) hematein. Figure S9: Logwood: MS//MS spectra of hc1, hc2, hc3, and hc4 acquired in negative ion mode. Figure S10: Sandalwood: MS/MS spectra of santalin-like compound acquired in negative ion mode. Table S1. Compounds and dyes identified in silk textiles dated to the 15th to 17th century.
