# Supplementary material for: A Mass Spectrometry-Based Approach for Characterization of Red, Blue, and Purple Natural Dyes
Source: Molecules. 2020 Jul 15;25(14):3223. doi: 10.3390/molecules25143223 (PMC7397139; doi:10.3390/molecules25143223)
Supplement: Supplementary file 1 [file molecules-25-03223-s001.pdf]

A mass spectrometry-based approach for characterization of red, blue, and purple natural dyes

Katarzyna Lech [1],* and Emilia Fornal [2]

[1] Warsaw University of Technology, Faculty of Chemistry, Noakowskiego 3, 00-664 Warsaw, Poland; klech@ch.pw.edu.pl
[2] Medical University of Lublin, Department of Pathophysiology, Jaczewskiego 8b, 20-090 Lublin, Poland; emilia.fornal@umlub.pl
* Correspondence: klech@ch.pw.edu.pl

Supplementary Materials

Figures and Table

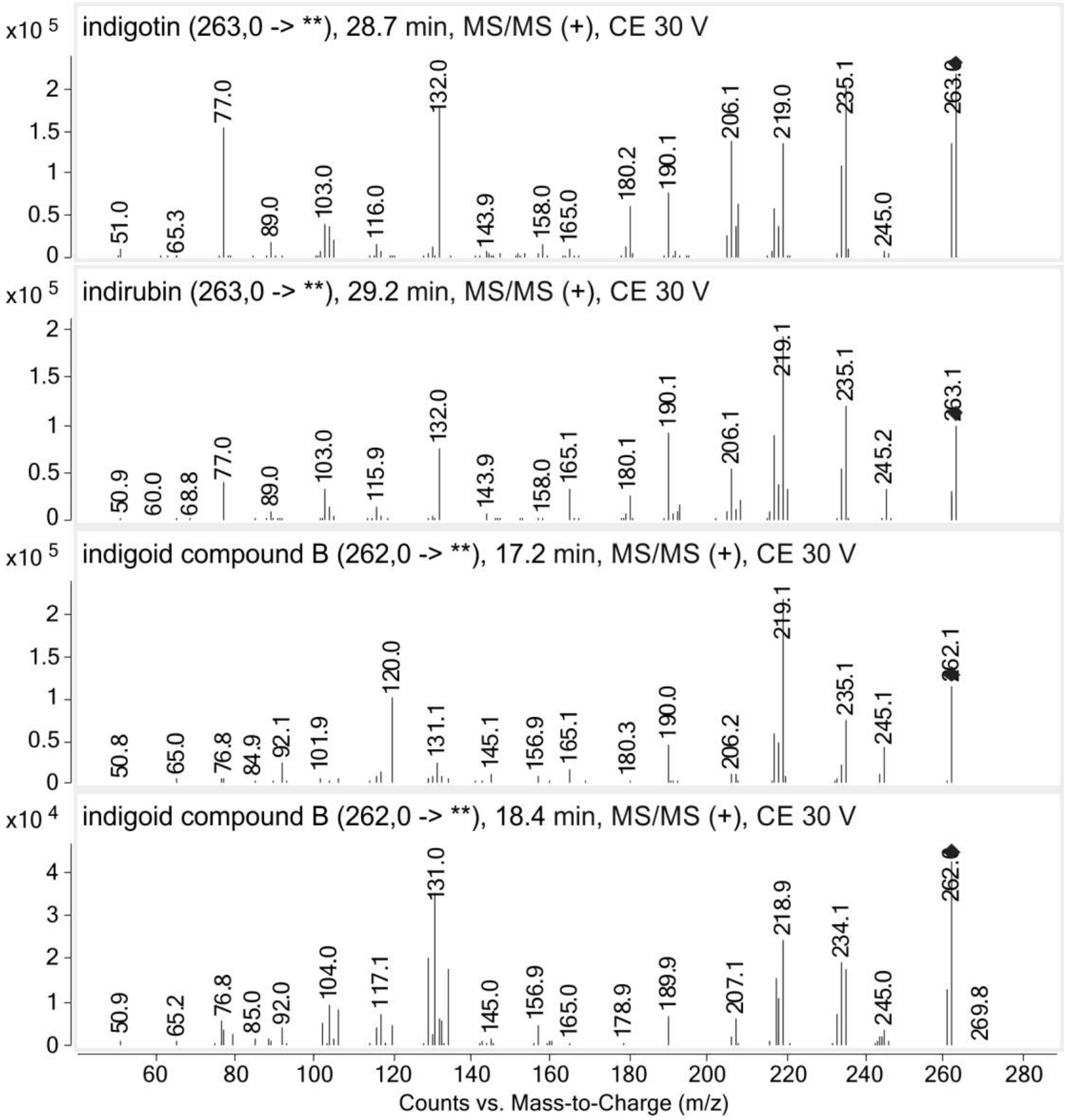

Figure S1. Indigo: MS/MS spectra of indigotin, indirubin, indigo compound A, and indigo compound A acquired in positive ion mode

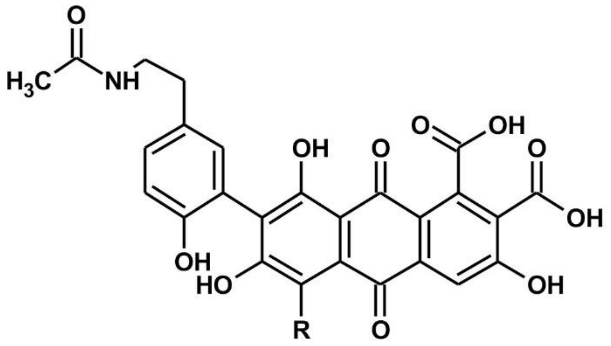

laccaic acid A, R = OH
xantholaccaic acid A, R = H

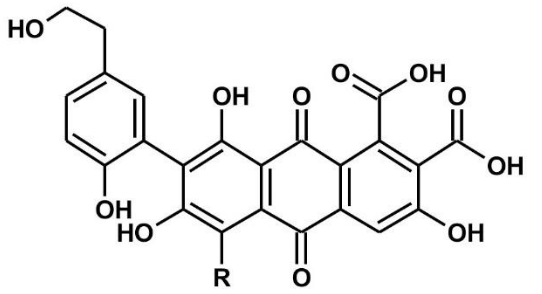

laccaic acid B, R = OH
xantholaccaic acid B, R = H

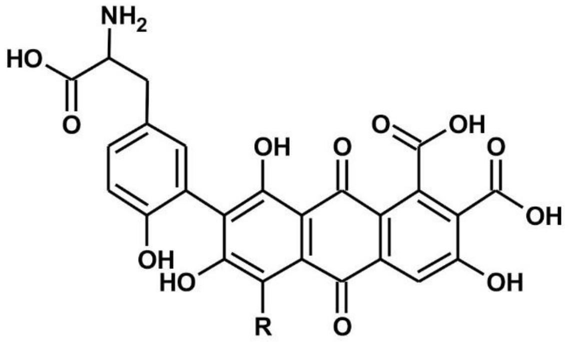

laccaic acid C, R = OH

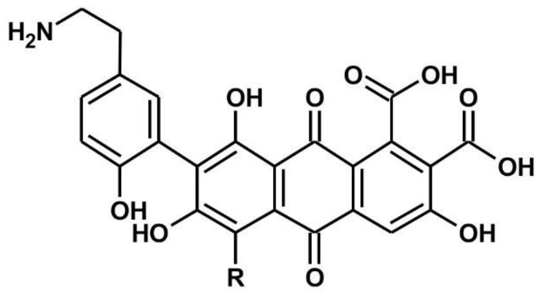

laccaic acid E, R = OH

Figure S2. Chemical structures of laccaic acids

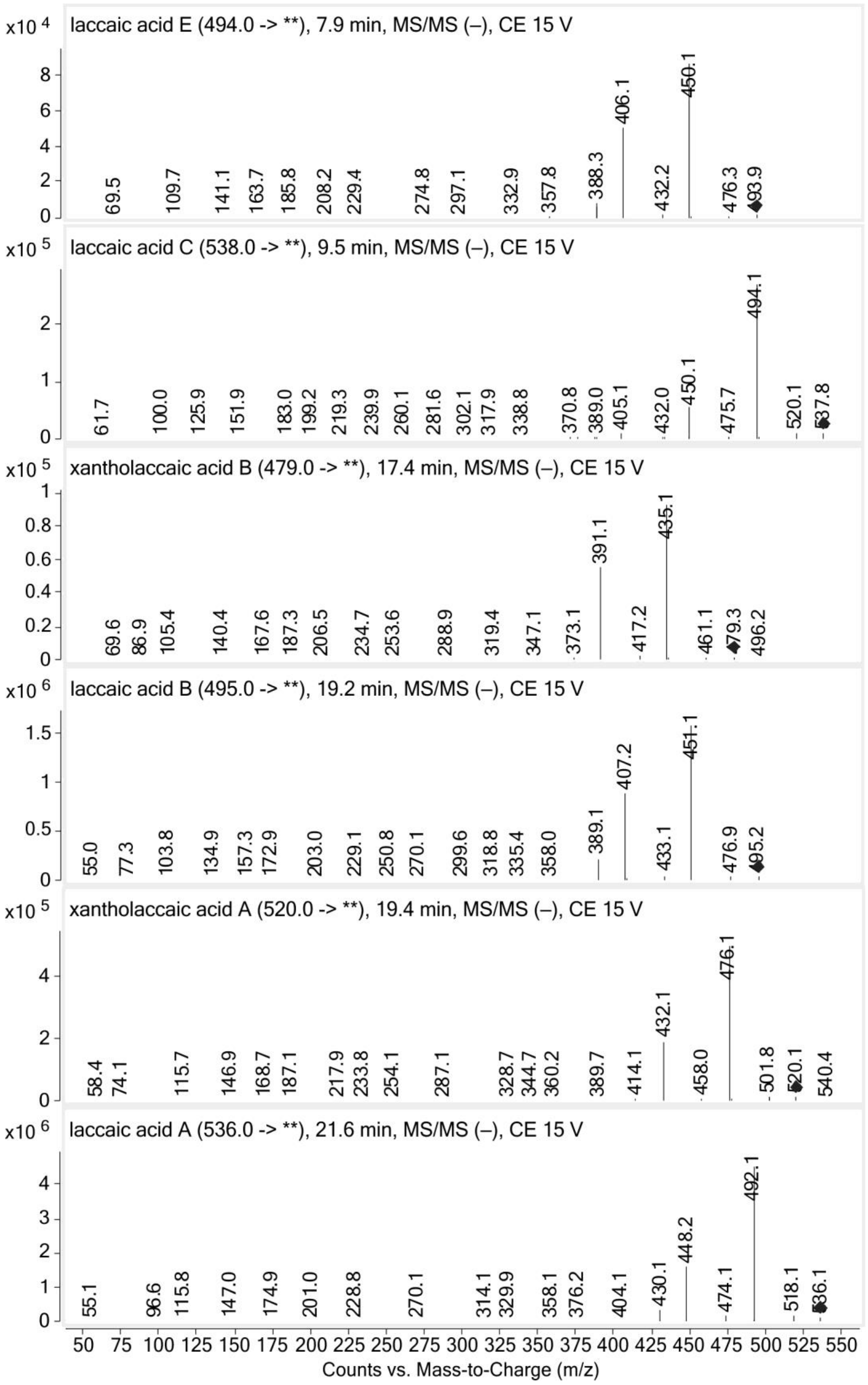

Figure S3. Lac dye: MS/MS spectra of laccaic acid E, laccaic acid C, xantholaccaic acid B, laccaic acid B, xantholaccaic acid A, and laccaic acid A, acquired in negative ion mode

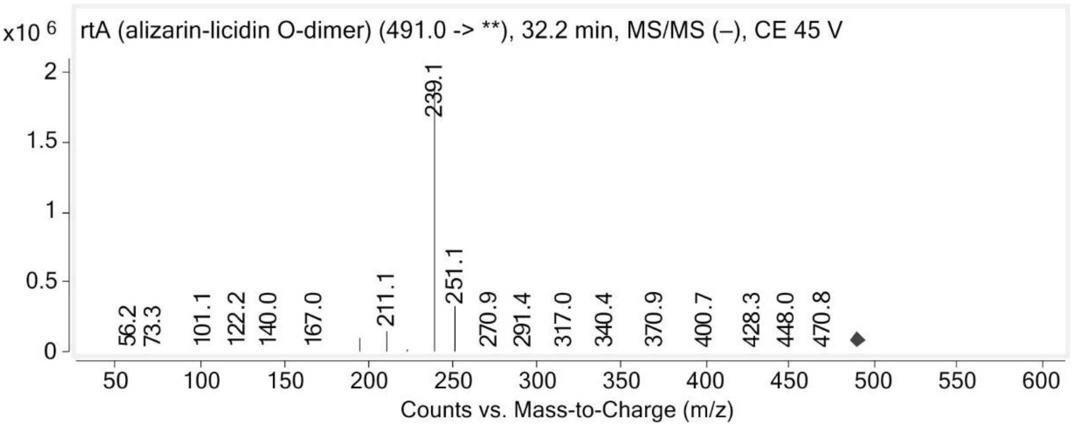

Figure S4. Madder: MS/MS spectra of rt1 (alizarin–lucidin dimer)

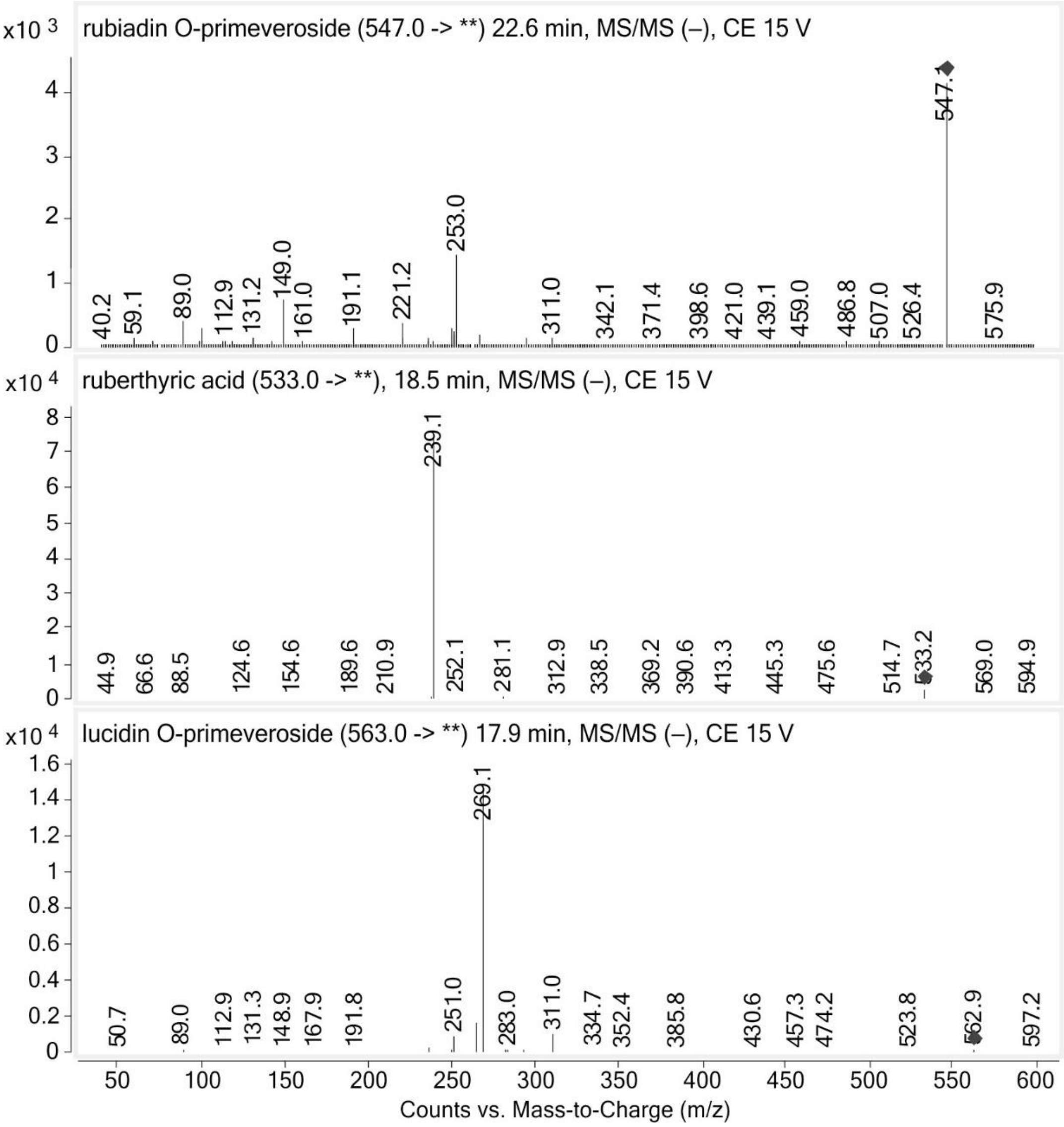

Figure S5. Madder: MS/MS spectra of rubiadin *O*-primeveroside, ruberthyric acid, and lucidin *O*-primeveroside acquired in negative ion mode

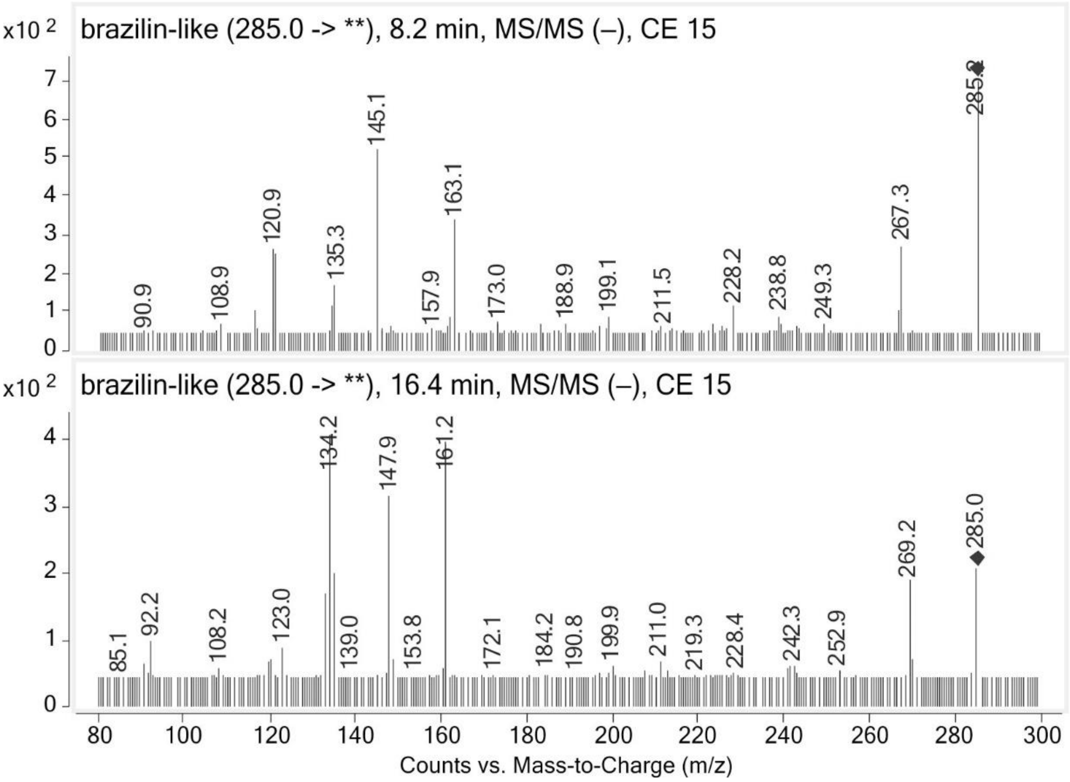

Figure S6. Brazilwood: MS/MS spectra of brazilin-like compounds in negative ion mode

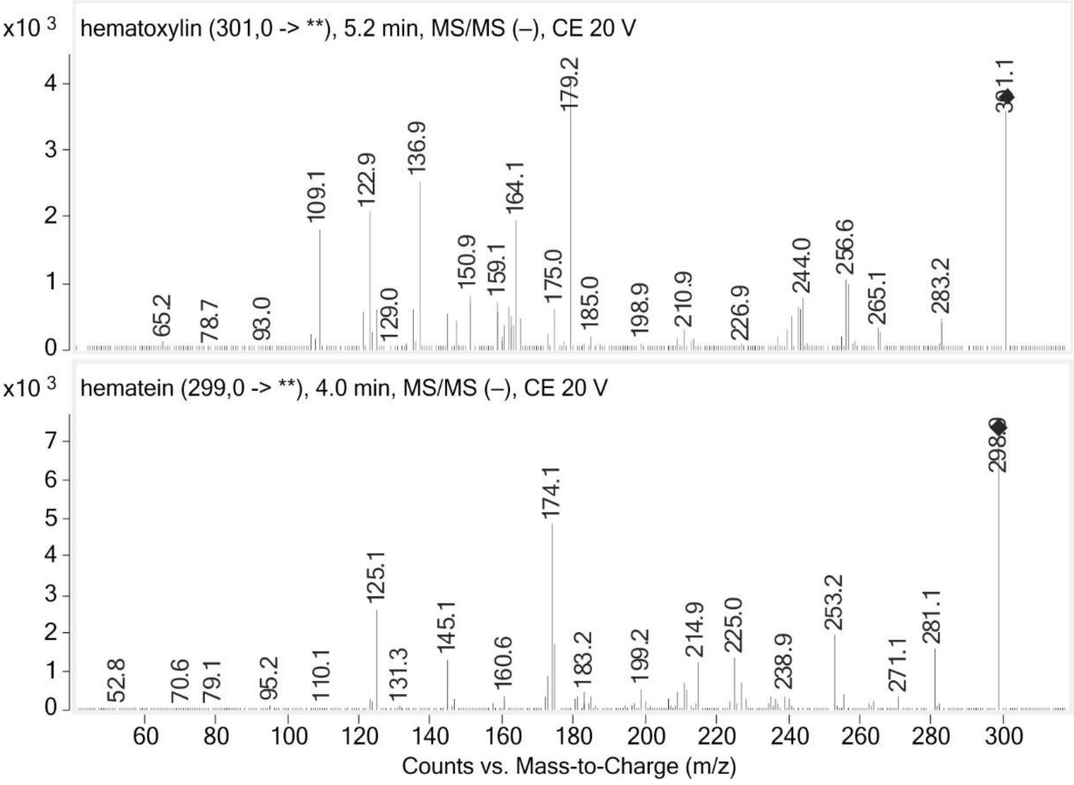

Figure S7. Logwood: MS/MS spectra of hematoxylin and hematein acquired in negative ion mode

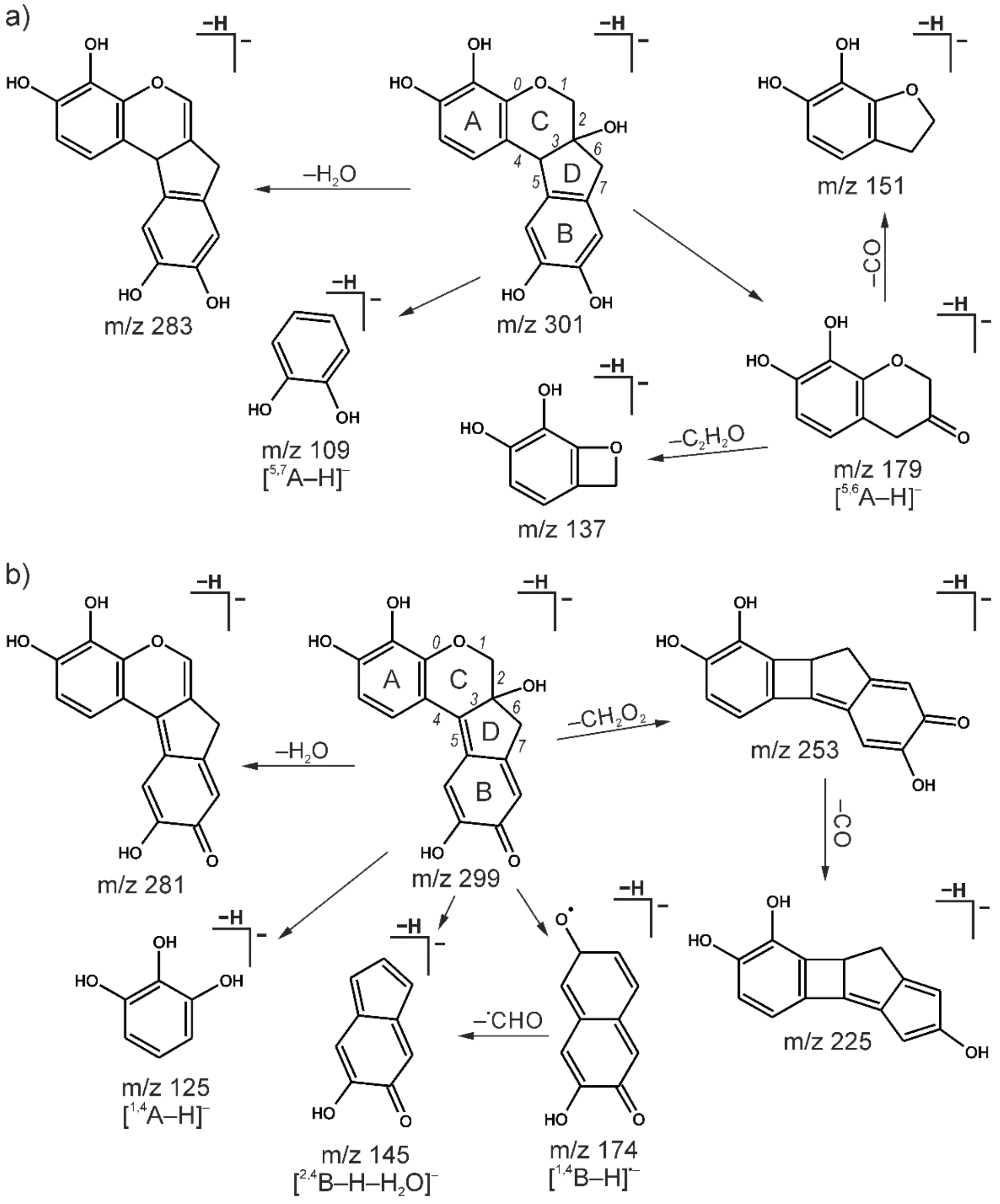

Figure S8. Proposed fragmentation pathways for a) hematoxylin and b) hematein

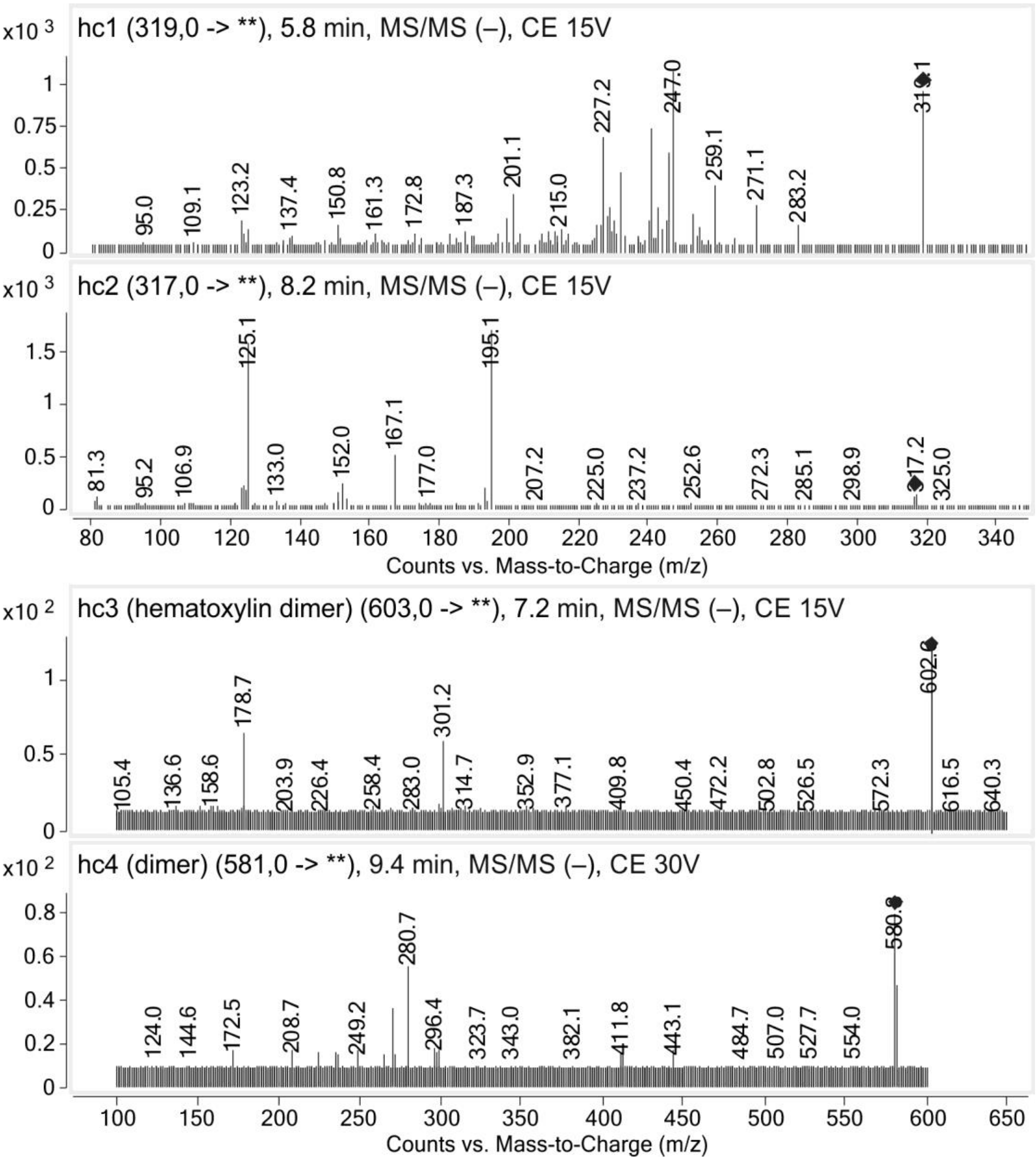

Figure S9. Logwood: MS//MS spectra of hc1, hc2, hc3, and hc4 acquired in negative ion mode

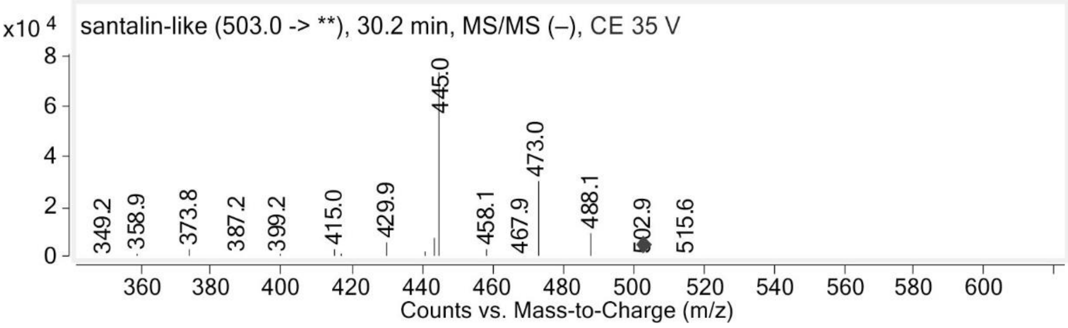

Figure S10. Sandalwood: MS/MS spectra of santalin-like compound acquired in negative ion mode

Table S1. Compounds and dyes identified in silk textiles dated to the 15th to 17th century.

Church	Textile number*	Object*	Origin and dating*	Fiber color	Identified compounds	Original dye	Remarks
Church of St Michael the Archangel and St Stanislaus Bishop and Martyr (Monastery Church of the Pauline Fathers)	No. 5	chasuble, part of back orphrey and around-neck-opening textile	Europe, the 15th cent. [1]	red	pp3, **<u>carminic acid</u>**, carminic acid derivative, ellagic acid, dcIV, dcVII, pp12, flavokermesic acid, kermesic acid, pp14, pp15	Polish or Armenian cochineal	
Church of St Michael the Archangel and St Stanislaus Bishop and Martyr (Monastery Church of the Pauline Fathers)	No. 5	chasuble, part of back orphrey and around-neck-opening textile	Europe, the 15th cent. [1]	red	laccaic acid E, **laccaic acid C**, carminic acid, xantholaccaic acid B, **<u>laccaic acid B</u>**, **<u>laccaic acid A</u>**, flavokermesic acid	lac dye	
Basilica of Holy Trinity (Monastery Church of the Dominicans)	No. 7	stole, main textile	Europe, the 15th cent. [1]	red	pp3, **<u>carminic acid</u>**, luteolin 7-*O*-glc, carminic acid derivative, ellagic acid, dcIV, dcVII, pp12, **flavokermesic acid**, **kermesic acid**, pp14, alizarin, pp15, purpurin	Polish or Armenian cochineal	
Basilica of Holy Trinity (Monastery Church of the Dominicans)	No. 9	cope, textile from mantle	Europe, the 15th cent. [1]	red	pp3, **<u>carminic acid</u>**, carminic acid derivative, ellagic acid, dcIV, dcVII, pp12, flavokermesic acid, kermesic acid, pp14, pp15	Polish or Armenian cochineal	
Basilica of Holy Trinity (Monastery Church of the Dominicans)	No. 13	dalmatic, front textile and insert in back orphrey	Europe, the 15th cent. [1]	red	ellagic acid, **flavokermesic acid**, **<u>kermesic acid</u>**, isatin, β/γ-aminoorcein, β/γ-aminoorceinimine, **α-aminoorcein**, β/γ-hydroxyorcein, **α-hydroxyorcein**	kermes + orchil	
Basilica of Holy Trinity (Monastery Church of the Dominicans)	No. 14	dalmatic, textile from back orphrey	Europe, the 15th cent. [1]	red	carminic acid, ellagic acid, **flavokermesic acid**, **<u>kermesic acid</u>**, isatin, β/γ-aminoorcein, β/γ-aminoorceinimine, **α-aminoorcein**, β/γ-hydroxyorcein, **α-hydroxyorcein**, indigotin	kermes + orchil	
Basilica of Holy Trinity (Monastery Church of the Dominicans)	No. 15	chasuble, textile from insert in right-front side	Europe, the 15th cent. [1]	red	dcII, **<u>carminic acid</u>**, carminic acid derivative, ellagic acid, dcIV, dcVII, flavokermesic acid, kermesic acid	American cochineal	
Church of St Francis of Assisi (Monastery Church of the Franciscans)	No. 16	stole, outer textile	Europe, the 16th cent. [1]	red	**flavokermesic acid**, **<u>kermesic acid</u>**, **α-aminoorcein**, α-hydroxyorcein	kermes + orchil	traces of synthetic dye

Church	Textile number*	Object*	Origin and dating*	Fiber color	Identified compounds	Original dye	Remarks
Corpus Christi Basilica	No. 17	chasuble, textile from sides	Europe, the 15th cent. [1]	red/purple	carminic acid, luteolin 7-*O*-glc, **flavokermesic acid**, **kermesic acid**, β/γ-aminoorcein, β/γ-aminoorceinimine, **β/γ-aminoorcein**, ***α*-aminoorcein**, β/γ-hydroxyorcein, *α*-hydroxyorcein	kermes + orchil	traces of synthetic dye
Church of the Divine Mercy	No. 18	chasuble, textile from back orphrey	Europe, the 15th cent. [1]	purple	dcII, **carminic acid**, genistin, luteolin 7-*O*-glc, carminic acid derivative, ellagic acid, dcIV, luteolin, genistein, luteolin methyl ether (1), flavokermesic acid, kermesic acid, biochanin A, isatin, β/γ-aminoorceinimine, β/γ-aminoorcein, ***α*-aminoorcein**, *α*-hydroxyorcein, **indigotin**, indirubin	indigo/woad + orchil + American cochineal + traces of dyer's broom	
Church of the Divine Mercy	No. 18	chasuble, textile from bottom part of front orphrey	Europe, the 15th cent. [1]	purple	luteolin *C*-hex, carminic acid, **genistin**, **luteolin 7-*O*-glc**, genistein *O*-hex, luteolin *O*-hex (2), **luteolin**, **genistein**, luteolin methyl ether (1), apigenin, biochanin A, isatin, β/γ-aminoorcein, β/γ-aminoorceinimine, β/γ-aminoorcein, ***α*-aminoorcein**, β/γ-hydroxyorcein, *α*-hydroxyorcein, **indigotin**, indirubin	indigo/woad + orchil + dyer's broom	
Basilica of Holy Trinity (Monastery Church of the Dominicans)	No. 20	chasuble, orphrey and around-neck-opening textile	Europe, the 16th cent. [1]	red	carminic acid, ellagic acid, **flavokermesic acid**, **kermesic acid**, β/γ-aminoorcein, β/γ-aminoorceinimine, β/γ-aminoorcein, ***α*-aminoorcein**, β/γ-hydroxyorcein, ***α*-hydroxyorcein**	kermes + orchil	
Church of the Divine Mercy	No. 21	stole, outer textile	Europe, the 15th cent. [1]	red	laccaic acid E, **laccaic acid C**, pp6, xantholaccaic acid B, **laccaic acid B**, **laccaic acid A**, flavokermesic acid, isatin, **indigotin**, indirubin	lac dye + traces of indigo/woad	
Church of the Divine Mercy	No. 22	chasuble, textile from middle part of front orphrey	Europe, the 15th cent. [1]	red	laccaic acid E, **laccaic acid C**, pp6, xantholaccaic acid B, **laccaic acid B**, **laccaic acid A**, flavokermesic acid, alizarin, purpurin, isatin, **indigotin**	lac dye + indigo/woad	
Church of the Divine Mercy	No. 23	chasuble, textile from upper part of back orphrey	Europe, the 15th-16th cent. [1]	red	laccaic acid E, **laccaic acid C**, pp6, xantholaccaic acid B, **laccaic acid B**, **laccaic acid A**, flavokermesic acid, alizarin, purpurin, isatin, **indigotin**	lac dye + traces of indigo/woad	

Church	Textile number*	Object*	Origin and dating*	Fiber color	Identified compounds	Original dye	Remarks
Church of the Divine Mercy	No. 24	chasuble, textile from back sides	Europe, the 15th-16th cent. [1]	red	laccaic acid E, **laccaic acid C**, pp6, xantholaccaic acid B, **laccaic acid B**, **laccaic acid A**, flavokermesic acid, alizarin, purpurin, isatin, **indigotin**	lac dye + traces of indigo/woad	
Church of the Divine Mercy	No. 25	maniple, textile from endings	Europe, the 15th-16th cent. [1]	red	laccaic acid E, **laccaic acid C**, pp6, xantholaccaic acid B, **laccaic acid B**, **laccaic acid A**, flavokermesic acid, alizarin, purpurin, isatin, **indigotin**	lac dye + indigo/woad	
Church of St Michael the Archangel and St Stanislaus Bishop and Martyr (Monastery Church of the Pauline Fathers)	No. 27	chasuble, orphrey and around-neck-opening textile	Europe, the 15th-16th cent. [1]	red	laccaic acid E, **laccaic acid C**, pp6, xantholaccaic acid B, **laccaic acid B**, **laccaic acid A**, flavokermesic acid, alizarin, purpurin, nordamnacanthal	lac dye	
Basilica of Holy Trinity (Monastery Church of the Dominicans)	No. 28	stole, main textile	Europe, the 15th-16th cent. [1]	red	laccaic acid E, laccaic acid C, pp6, xantholaccaic acid B, **laccaic acid B**, **laccaic acid A**, flavokermesic acid, isatin, indigotin, indirubin	lac dye + traces of indigo/woad	
Basilica of Holy Trinity (Monastery Church of the Dominicans)	No. 29	chasuble, textile from sides	Europe, the 15th-16th cent. [1]	red	pp3, **carminic acid**, carminic acid derivative, **ellagic acid**, dcIV, dcVII, pp12, flavokermesic acid, kermesic acid, pp14, pp15	Polish or Armenian cochineal + traces of indigo/woad	presence of synthetic dye, original fiber color - red
Church of the Assumption of the Blessed Virgin Mary (Monastery Church of the Camaldolese Monks)	No. 30	chasuble, textile from sides	Europe, the 16th cent. [1]	purple	carminic acid, isatin, β/γ-aminoorcein, β/γ-aminoorceinimine, β/γ-aminoorcein, **α-aminoorcein**, **β/γ-hydroxyorcein**, **α-hydroxyorcein**, **indigotin**	indigo/woad + orchil	
Church of St Francis of Assisi (Monastery Church of the Franciscans)	No. 33	chasuble, main textile	Europe, the 16th cent. [1]	red	carminic acid, luteolin di-*O*-hex, luteolin 7-*O*-glc, apigenin 7-*O*-glc, luteolin, isatin, β/γ-aminoorcein, β/γ-aminoorceinimine, **β/γ-aminoorcein**, **α-aminoorcein**, β/γ-hydroxyorcein, **α-hydroxyorcein**, **indigotin**	orchil + indigo/woad + traces of cochineal (of unknown origin) and probably weld	

Church	Textile number*	Object*	Origin and dating*	Fiber color	Identified compounds	Original dye	Remarks
Church of St Catherine of Alexandria	No. 34	cope, main textile	Europe, the 16th cent. [1]	red	dcII, **carminic acid**, dc*O*fka, carminic acid derivative, **ellagic acid**, dcIV, dcVII, flavokermesic acid, kermesic acid	American cochineal	
Basilica of Holy Trinity (Monastery Church of the Dominicans)	No. 35	chasuble, textile from sides	Europe, the 16th cent. [1]	red	dcII, **carminic acid**, carminic acid derivative, ellagic acid, dcIV, dcVII, flavokermesic acid, kermesic acid	American cochineal	traces of synthetic dye
Church of St Mark	No. 36	chasuble, main textile	Europe, the 16th cent. [1]	red	pp3, **carminic acid**, carminic acid derivative, ellagic acid, dcIV, dcVII, pp12, flavokermesic acid, **kermesic acid**, pp14, pp15	Polish or Armenian cochineal	
Basilica of Holy Trinity (Monastery Church of the Dominicans)	No. 38	stole, burse and maniple, main textile	Europe, the 16th cent. [1]	red	digalloylglucose, trigalloylglucose, tetragalloylglucose (1), tetragalloylglucose (2), dcII, **carminic acid**, dc*O*fka, carminic acid derivative, **ellagic acid**, dcIV, dcVII, flavokermesic acid, kermesic acid	American cochineal	
Church of St Stephen	No. 38	chasuble, main textile	Europe, the 16th cent. [1]	red	dcII, **carminic acid**, carminic acid derivative, ellagic acid, dcIV, dcVII, flavokermesic acid	American cochineal	
Corpus Christi Basilica	No. 41	chasuble, textile from back orphrey	Europe, the 16th cent. [1]	red	dcII, **carminic acid**, luteolin 7-*O*-glc, carminic acid derivative, **ellagic acid**, dcIV, dcVII, luteolin, flavokermesic acid, kermesic acid	American cochineal	
Church of St Stephen	No. 42	chalice velum and pall, outer textile	Europe, the 16th cent. [1]	red	digalloylglucose, dcII, **carminic acid**, carminic acid derivative, ellagic acid, dcIV, dcVII, flavokermesic acid, kermesic acid	American cochineal	
Corpus Christi Basilica	No. 44	chasuble, textile from sides	Europe, the 16th cent. [1]	red	digalloylglucose, trigalloylglucose, **dcII**, **carminic acid**, dc*O*fka, carminic acid derivative, **ellagic acid**, dcIV, dcVII, flavokermesic acid, kermesic acid	American cochineal + ellagitannins	
Church of the Conversion of St Paul (Monastery Church of the Lazarists)	No. 45	chasuble, main textile	Europe, the 16th cent. [1]	red	dcII, **carminic acid**, carminic acid derivative, ellagic acid, dcIV, dcVII, flavokermesic acid, kermesic acid	American cochineal	
Church of St Francis of Assisi (Monastery Church of the Franciscans)	No. 46	chasuble, textile from sides on back	Europe, the 16th cent. [1]	red	dcII, **carminic acid**, carminic acid derivative, ellagic acid, dcIV, dcVII, flavokermesic acid, kermesic acid	American cochineal	

Church	Textile number*	Object*	Origin and dating*	Fiber color	Identified compounds	Original dye	Remarks
Basilica of Holy Trinity (Monastery Church of the Dominicans)	No. 47	maniple, main textile	Europe, the 16th cent. [1]	red	dcII, **carminic acid**, luteolin 7-*O*-glc, carminic acid derivative, ellagic acid, dcIV, dcVII, flavokermesic acid, kermesic acid	American cochineal	
Basilica of Holy Trinity (Monastery Church of the Dominicans)	No. 49	stole, main textile	Europe, the 16th cent. [1]	red	dcII, **carminic acid**, dc*O*fka, carminic acid derivative, ellagic acid, dcIV, **dcVII**, flavokermesic acid, kermesic acid	American cochineal	
Basilica of Holy Trinity (Monastery Church of the Dominicans)	No. 49	dalmatic, main textile	Europe, the 16th cent. [1]	red	dcII, **carminic acid**, dc*O*fka, carminic acid derivative, ellagic acid, dcIV, **dcVII**, flavokermesic acid, kermesic acid	American cochineal	
Church of St Francis of Assisi (Monastery Church of the Franciscans)	No. 50	chasuble, textile from sides on front	Europe, the 16th cent. [1]	red	dcII, **carminic acid**, dc*O*fka, carminic acid derivative, ellagic acid, dcIV, dcVII, flavokermesic acid, kermesic acid	American cochineal	
Church of the Assumption of the Blessed Virgin Mary (Monastery Church of the Camaldolese Monks)	No. 51	chasuble, main textile	Europe, the 16th cent. [1]	red	digalloylglucose, trigalloylglucose, **tetragalloylglucose (1)**, tetragalloylglucose (2), pentagalloylglucose, dcII, **carminic acid**, carminic acid derivative, **ellagic acid**, dcIV, dcVII, flavokermesic acid, kermesic acid	American cochineal + ellagitannins	
Church of St Andrew (Monastery Church of the Poor Clares)	No. 51	maniple, main textile	Europe, the 16th cent. [1]	red	digalloylglucose, trigalloylglucose, tetragalloylglucose (1), dcII, **carminic acid**, dc*O*fka, carminic acid derivative, **ellagic acid**, dcIV, dcVII, flavokermesic acid	American cochineal + ellagitannins	
Basilica of Holy Trinity (Monastery Church of the Dominicans)	No. 53	stole, main textile	Europe, the 15th cent. [1]	red	dcII, **carminic acid**, luteolin 7-*O*-glc, dc*O*fka, carminic acid derivative, ellagic acid, dcIV, dcVII, flavokermesic acid, kermesic acid	American cochineal	
Church of St Francis of Assisi (Monastery Church of the Franciscans)	No. 55	chasuble, main textile	Europe, the 16th cent. [1]	blue	isatin, *α*-aminoorcein, **indigotin**, indirubin	indigo/woad	
Church of St Francis of Assisi (Monastery Church of the Franciscans)	No. 56	chasuble, textile from insert in front sides	Europe, the 16th cent. [1]	blue	isatin, indigoid compound A, *α*-aminoorcein, **indigotin**, indirubin	indigo/woad	
Church of St Francis of Assisi (Monastery Church of the Franciscans)	No. 57	stole and maniple, outer textile	Europe, the 16th cent. [1]	blue	**luteolin di-*O*-hex**, **luteolin 7-*O*-glc**, apigenin 7-*O*-glc, luteolin *O*-hex (2), sulfuretin, **luteolin**, apigenin, diosmetin, isatin, β/γ-aminoorcein, ***α*-**	indigo/woad + weld + traces of orchil	

Church	Textile number*	Object*	Origin and dating*	Fiber color	Identified compounds	Original dye	Remarks
					**aminoorcein**, α-hydroxyorcein, **<u>indigotin</u>**, indirubin		
Church of St Francis of Assisi (Monastery Church of the Franciscans)	No. 58	stole, main textile	Europe, the 16th cent. [1]	yellow	digalloylglucose, brazilein, **protosappanin B**, **caes1**, **<u>caesD</u>**, luteolin di-*O*-hex, **<u>urolithin C</u>**, **<u>luteolin 7-*O*-glc</u>**, ellagic acid, apigenin 7-*O*-glc, luteolin *O*-hex, luteolin	brazilwood + weld + ellagitannins	
Basilica of Holy Trinity (Monastery Church of the Dominicans)	No. 60	chasuble, around-neck-opening textile	Europe, the 16th cent. [1]	red	carminic acid, **flavokermesic acid**, **<u>kermesic acid</u>**, β/γ-aminoorcein, β/γ-aminoorceinimine, **<u>α-aminoorcein</u>**, **α-hydroxyorcein**	kermes + orchil	
Basilica of Holy Trinity (Monastery Church of the Dominicans)	No. 60	chasuble, textile from sides on back	Europe, the 16th cent. [1]	red	dcII, **<u>carminic acid</u>**, carminic acid derivative, ellagic acid, dcIV, dcVII, flavokermesic acid, kermesic acid	American cochineal	traces of synthetic dyes
Church of St Peter and St Paul	No. 61	chasuble, textile from some parts of sides	Europe, the 16th cent. [1]	red	digalloylglucose, trigalloylglucose, **<u>carminic acid</u>**, carminic acid derivative, **ellagic acid**, dcIV, dcVII, flavokermesic acid, kermesic acid	American cochineal + ellagitannins	presence of synthetic dye, original fiber color - red
Corpus Christi Basilica	No. 62	chasuble, textile from orphrey and right arm on back	Europe, the 16th cent. [1]	red	**digalloylglucose**, **trigalloylglucose**, **tetragalloylglucose (1)**, tetragalloylglucose (2), dcII, **<u>carminic acid</u>**, dc*O*fka, carminic acid derivative, **ellagic acid**, dcIV, dcVII, flavokermesic acid, kermesic acid,	American cochineal + ellagitannins	
Corpus Christi Basilica	No. 63	chasuble, textile from inserts of front sides	Europe, the 16th cent. [1]	red	digalloylglucose, dcII, **<u>carminic acid</u>**, dc*O*fka, carminic acid derivative, **ellagic acid**, dcIV, dcVII, flavokermesic acid, kermesic acid	American cochineal	traces of synthetic dye
Church of St Mark	No. 63	burse, main textile	Europe, the 16th cent. [1]	red	**<u>carminic acid</u>**, carminic acid derivative, ellagic acid, dcIV, dcVII	American cochineal	
Church of St Francis of Assisi (Monastery Church of the Franciscans)	No. 66	chasuble, main textile	Europe, the 16th cent. [1]	red	carminic acid, **luteolin di-*O*-hex**, **luteolin 7-*O*-glc**, apigenin 7-*O*-glc, luteolin *O*-hex (2), luteolin, apigenin, diosmetin, isatin, β/γ-aminoorcein, β/γ-aminoorceinimine, **β/γ-aminoorcein**, **<u>α-aminoorcein</u>**, β/γ-hydroxyorcein, **α-hydroxyorcein**, **<u>indigotin</u>**	orchil + weld + indigo/woad	

Church	Textile number*	Object*	Origin and dating*	Fiber color	Identified compounds	Original dye	Remarks
Basilica of Holy Trinity (Monastery Church of the Dominicans)	No. 67	cope, textile from orphrey and hood	Europe, the 15th cent. [1]	red	dcII, **carminic acid**, carminic acid derivative, ellagic acid, dcIV, dcVII, flavokermesic acid, kermesic acid	American cochineal	
Corpus Christi Basilica	No. 68	chasuble, textile from orphrey and back sides	Europe, the 16th cent. [1]	red	dcII, **carminic acid**, luteolin 7-*O*-glc, carminic acid derivative, ellagic acid, dcIV, dcVII, flavokermesic acid, kermesic acid	American cochineal	
Basilica of Holy Trinity (Monastery Church of the Dominicans)	No. 71	parura, lining	Europe, the 16th-17th cent. [1]	red	dcII, **carminic acid**, carminic acid derivative, ellagic acid, dcIV, dcVII, flavokermesic acid, kermesic acid	American cochineal	
Basilica of Holy Trinity (Monastery Church of the Dominicans)	No. 73	stole, textile from band	Europe, the 16th cent. [1]	red	**carminic acid**, carminic acid derivative, **ellagic acid**, dcIV, dcVII, kermesic acid	American cochineal	presence of synthetic dye, original fiber color - red
Basilica of Holy Trinity (Monastery Church of the Dominicans)	No. 73	chalice velum, main textile	Europe, the 16th cent. [1]	red	**carminic acid**, carminic acid derivative, ellagic acid, dcIV, dcVII	American cochineal	presence of synthetic dye, original fiber color - red
Church of St Andrew (Monastery Church of the Poor Clares)	No. 74	maniple, outer textile	Europe, the 16th cent. [1]	red	dcII, **carminic acid**, carminic acid derivative, dcIV, dcVII	American cochineal	
Church of St Adalbert	No. 75	maniple, outer textile	Europe, the 16th cent. [1]	red	pp3, **carminic acid**, carminic acid derivative, ellagic acid, dcIV, dcVII, pp12, flavokermesic acid, **kermesic acid**, pp14, alizarin, pp15, purpurin	Polish or Armenian cochineal + traces of *Rubiaceae* dye plant (probably *Galium* species)	

Church	Textile number*	Object*	Origin and dating*	Fiber color	Identified compounds	Original dye	Remarks
Basilica of Holy Trinity (Monastery Church of the Dominicans)	No. 76	chasuble, textile from sides	Europe, the 16th cent. [1]	red	dcII, **carminic acid**, carminic acid derivative, **ellagic acid**, dcIV, dcVII, flavokermesic acid, kermesic acid	American cochineal	traces of synthetic dyes
Corpus Christi Basilica	No. 78	chasuble, textile from inserts of front sides	Europe, the 16th cent. [1]	red	**flavokermesic acid**, **kermesic acid**, β/γ-aminoorcein, β/γ-aminoorceinimine, **β/γ-aminoorcein**, **α-aminoorcein**, β/γ-hydroxyorcein, **α-hydroxyorcein**	kermes + orchil	traces of synthetic dye
Corpus Christi Basilica	No. 81	chalice velum, outer textile	Europe, the 16th cent. [1]	red	digalloylglucose, caesD, dcII, **carminic acid**, dc*O*fka, carminic acid derivative, ellagic acid, dcIV, dcVII, flavokermesic acid, kermesic acid	American cochineal + ellagitannins	traces of synthetic dyes
Church of St Mark	No. 82	chasuble, textile from front sides and on back	Europe, the 16th cent. [1]	red	digalloylglucose, dcII, **carminic acid**, carminic acid derivative, ellagic acid, dcIV, dcVII, flavokermesic acid	American cochineal + ellagitannins	
Church of St Stephen	No. 83	chasuble, main textile	Europe, the 16th cent. [1]	red	dcII, **carminic acid**, carminic acid derivative, ellagic acid, dcIV, dcVII, flavokermesic acid, kermesic acid	American cochineal	
Church of St Francis of Assisi (Monastery Church of the Franciscans)	No. 85	chasuble, textile from orphrey	Europe, the 16th cent. [1]	red	dcII, **carminic acid**, carminic acid derivative, **ellagic acid**, dcIV, dcVII, flavokermesic acid, kermesic acid	American cochineal	
Church of St Francis of Assisi (Monastery Church of the Franciscans)	No. 86	stole, outer textile	Europe, the 16th cent. [1]	red	dcII, **carminic acid**, carminic acid derivative, ellagic acid, dcIV, dcVII, flavokermesic acid, kermesic acid	American cochineal	
Church of the Visitation of the Blessed Virgin Mary (Monastery Church of the Carmelites)	No. 93	chasuble, textile from sides	N/A, the 17th cent. [2]	red/ /purple	dcII, **carminic acid**, carminic acid derivative, **ellagic acid**, dcIV, dcVII, flavokermesic acid	American cochineal	
Church of St Joseph (Monastery Church of the Bernardine Nuns)	No. 97	chalice velum and burse, outer textile	Europe, the 17th cent. [2]	red	dcII, **carminic acid**, dcOfka, carminic acid derivative, ellagic acid, dcIV, dcVII, flavokermesic acid, kermesic acid	American cochineal	
Basilica of Holy Trinity (Monastery Church of the Dominicans)	No. 98	bandera with the vision of St Jack, insert in pelmet	Europe, the 17th cent.[2]	red	**carminic acid**, carminic acid derivative, ellagic acid, dcIV, dcVII	cochineal (of unknown origin)	
Church of the Visitation of the Blessed Virgin Mary	No. 99	chasuble, main textile	N/A, the 17th cent. [2]	red/ /purple	dcII, **carminic acid**, carminic acid derivative, dcIV, dcVII	American cochineal	

Church	Textile number*	Object*	Origin and dating*	Fiber color	Identified compounds	Original dye	Remarks
(Monastery Church of the Carmelites)							
Church of St Peter and St Paul	No. 103	maniple, main textile	Europe, the 17th cent.	red	digalloylglucose, dcII, **<u>carminic acid</u>**, dcOfka, carminic acid derivative, ellagic acid, dcIV, dcVII, flavokermesic acid, kermesic acid	American cochineal + ellagotannins + traces of indigo/woad	traces of synthetic dye
Basilica of Holy Trinity (Monastery Church of the Dominicans)	No. 104	bandera with the vision of St Jack, background textile	Europe, the 17th cent.	red	**<u>carminic acid</u>**, carminic acid derivative, ellagic acid, dcIV, dcVII	cochineal (of unknown origin)	
Church of St Francis of Assisi (Monastery Church of the Franciscans)	No. 108	chasuble, textile from front orphrey	Europe, the 17th cent.	red	dcII, **<u>carminic acid</u>**, dcOfka, carminic acid derivative, **ellagic acid**, dcIV, dcVII, flavokermesic acid, kermesic acid	American cochineal	
Church of the Assumption of the Blessed Virgin Mary (Monastery Church of the Camaldolese Monks)	No. 111	chasuble, main textile	Europe, the 17th cent.	purple	**hematein**, caesD, carminic acid, **caesC**, isatin, β/γ-aminoorcein, β/γ-aminoorceinimine, β/γ-aminoorcein, **α-aminoorcein**, β/γ-hydroxyorcein, **α-hydroxyorcein**, **<u>indigotin</u>**, indirubin	indigo/woad + orchil + traces of logwood	
Basilica of Holy Trinity (Monastery Church of the Dominicans)	No. 115	chasuble, textile from sides	Europe, the 17th cent. [2]	red	**dcII**, **<u>carminic acid</u>**, dcOfka, carminic acid derivative, ellagic acid, dcIV, dcVII, flavokermesic acid, kermesic acid	American cochineal	traces of synthetic dyes
Church of St Stephen	No. 117	chasuble, textile from sides	Europe, the 17th cent. [2]	red	dcII, **<u>carminic acid</u>**, carminic acid derivative, ellagic acid, dcIV, dcVII	American cochineal	
Church of St Casimir the Prince (Monastery Church of the Reformed Franciscans)	No. 117	chasuble, textile from sides	Europe, the 17th cent. [2]	red	**<u>carminic acid</u>**, carminic acid derivative, dcIV, dcVII, kermesic acid	American cochineal	
Basilica of Holy Trinity (Monastery Church of the Dominicans)	No. 119	chasuble, textile from back orphrey	Europe, the 17th cent. [2]	red	**dcII**, **<u>carminic acid</u>**, carminic acid derivative, ellagic acid, dcIV, dcVII, flavokermesic acid, kermesic acid	American cochineal	
Basilica of Holy Trinity (Monastery Church of the Dominicans)	No. 119	chasuble, back orphrey and around-neck-opening textile	Europe, the 17th cent. [2]	red	**dcII**, **<u>carminic acid</u>**, dcOfka, carminic acid derivative, ellagic acid, dcIV, dcVII, **flavokermesic acid**, kermesic acid	American cochineal	

Church	Textile number*	Object*	Origin and dating*	Fiber color	Identified compounds	Original dye	Remarks
Church of St Francis of Assisi (Monastery Church of the Franciscans)	No. 121	chasuble, textile from back orphrey	Europe, the 17th cent. [2]	red	digalloylglucose, trigalloylglucose, tetragalloylglucose (1), caesD, dcII, <u>**carminic acid**</u>, caesC, dcOfka, carminic acid derivative, **ellagic acid**, dcIV, dcVII, flavokermesic acid, kermesic acid	American cochineal + traces of brazilwood	
Church of St Francis of Assisi (Monastery Church of the Franciscans)	No. 122	chasuble, textile from sides and around-neck-opening	Europe, the 17th cent. [2]	red	dcII, <u>**carminic acid**</u>, dcOfka, carminic acid derivative, **ellagic acid**, dcIV, dcVII, flavokermesic acid, kermesic acid	American cochineal	
Church of St Francis of Assisi (Monastery Church of the Franciscans)	No. 123	chasuble, textile from front, back sides and around-neck-opening	Europe, the 17th cent. [2]	red	dcII, <u>**carminic acid**</u>, dcOfka, carminic acid derivative, **ellagic acid**, dcIV, dcVII, flavokermesic acid, kermesic acid	American cochineal	
Church of the Assumption of the Holy Mother and St Venceslaus (Monastery Church of the Cistercians)	No. 124	chasuble, main textile	Europe, the 17th cent. [2]	red	dcII, <u>**carminic acid**</u>, carminic acid derivative, **ellagic acid**, dcIV, dcVII, flavokermesic acid, kermesic acid, isatin, indigotin	American cochineal + traces of indigo/woad	
Church of St Francis of Assisi (Monastery Church of the Franciscans)	No. 124	chasuble, textile from back orphrey	Europe, the 17th cent. [2]	red	dcII, <u>**carminic acid**</u>, dcOfka, carminic acid derivative, **ellagic acid**, dcIV, dcVII, flavokermesic acid, kermesic acid	American cochineal	
Church of St Joseph (Monastery Church of the Bernardine Nuns)	No. 125	chasuble, textile from sides	Europe, the 17th cent. [2]	red	dcII, <u>**carminic acid**</u>, carminic acid derivative, **ellagic acid**, dcIV, dcVII, flavokermesic acid, kermesic acid	American cochineal	
Church of St Mark	No. 126	chasuble, around-neck-opening textile	Europe, the 17th cent. [2]	red	digalloylglucose, dcII, <u>**carminic acid**</u>, carminic acid derivative, ellagic acid, dcIV, dcVII, flavokermesic acid	American cochineal	
Church of St Joseph (Monastery Church of the Bernardine Nuns)	No. 129	chasuble, textile from sides	Europe, the 17th cent. [2]	red	digalloylglucose, dcII, <u>**carminic acid**</u>, dcOfka, carminic acid derivative, ellagic acid, dcIV, dcVII, flavokermesic acid, kermesic acid	American cochineal + ellagotannins	
Basilica of Holy Trinity (Monastery Church of the Dominicans)	No. 130	chasuble, around-neck-opening textile	Europe, the 17th cent. [2]	red	<u>**genistin**</u>, <u>**luteolin 7-*O*-glc**</u>, genistein *O*-hex, luteolin *O*-hex (1), **apigenin 7-*O*-glc**, luteolin *O*-hex (2), **luteolin**, **genistin**, luteolin methyl ether (1), apigenin, biochanin A, isatin, <u>**indigotin**</u>, indirubin	American cochineal + traces of indigo/woad	

Church	Textile number*	Object*	Origin and dating*	Fiber color	Identified compounds	Original dye	Remarks
Church of St Andrew (Monastery Church of the Poor Clares)	No. 131	chasuble, piece of textile	Europe, the 17th cent. [2]	red	digalloylglucose, **dcII**, **carminic acid**, dcOfka, carminic acid derivative, ellagic acid, dcIV, dcVII, flavokermesic acid	American cochineal + ellagotannins	
Basilica of Holy Trinity (Monastery Church of the Dominicans)	No. 132	chasuble, front inserts in arms	Europe, the 17th cent. [2]	red	**dcII**, **carminic acid**, dcOfka, carminic acid derivative, ellagic acid, dcIV, dcVII, **flavokermesic acid**, kermesic acid	American cochineal	
Church of St Peter and St Paul	No. 135	chasuble, main textile	Europe, the 17th cent. [2]	red	dcII, **carminic acid**, dcOfka, carminic acid derivative, ellagic acid, dcIV, dcVII, flavokermesic acid, kermesic acid	American cochineal	
Corpus Christi Basilica	No. 136	chalice velum, outer textile	Europe, the 17th cent. [2]	blue with purple hue	dcII, **carminic acid**, luteolin di-*O*-hex, luteolin 7-*O*-glc, dcIV, dcVII, flavokermesic acid, kermesic acid, alizarin, xanthopurpurin, purpurin, isatin, β/γ-aminoorcein, β/γ-aminoorceinimine, β/γ-aminoorcein, ***α*-aminoorcein**, β/γ-hydroxyorcein, ***α*-hydroxyorcein**, **indigotin**	orchil + American cochineal + indigo/woad	presence of synthetic dyes, original fiber color - purple
Basilica of Holy Trinity (Monastery Church of the Dominicans)	No. 138	pall, main textile	Europe, the 17th cent. [2]	blue	isatin, β/γ-aminoorcein, *α*-aminoorcein, *α*-hydroxyorcein, isatin, **indigotin**, indirubin	indigo/woad + orchil	
Church of St Andrew (Monastery Church of the Poor Clares)	No. 141	chasuble, textile from sides	Europe, the 17th cent. [2]	red	digalloylglucose, trigalloylglucose, tetragalloylglucose (1), dcII, **carminic acid**, carminic acid derivative, **ellagic acid**, dcIV, dcVII	American cochineal + ellagotannins	
Church of St Andrew (Monastery Church of the Poor Clares)	No. 142	chasuble, textile from sides	Europe, the 17th cent. [2]	red	dcII, **carminic acid**, carminic acid derivative, dcIV, dcVII	American cochineal	
Basilica of Holy Trinity (Monastery Church of the Dominicans)	No. 144	chasuble, textile from orphrey and upper parts of front sides	Europe, the 17th cent. [2]	purple	dcII, **carminic acid**, carminic acid derivative, dcIV, dcVII, flavokermesic acid, kermesic acid, isatin, β/γ-aminoorcein, β/γ-aminoorceinimine, β/γ-aminoorcein, ***α*-aminoorcein**, β/γ-hydroxyorcein, ***α*-hydroxyorcein**, **indigotin**, indirubin	indigo/woad + American cochineal + orchil	
Basilica of Holy Trinity (Monastery Church of the Dominicans)	No. 144	burse and maniple, main textile	Europe, the 17th cent. [2]	blue	isatin, ***α*-aminoorcein**, ***α*-hydroxyorcein**, **indigotin**	indigo/woad + orchil	

Church	Textile number*	Object*	Origin and dating*	Fiber color	Identified compounds	Original dye	Remarks
Church of the Visitation of the Blessed Virgin Mary (Monastery Church of the Carmelites)	No. 147	chasuble, textile from sides	N/A, the 17th cent. [2]	red	caesD, dcII, **carminic acid**, **caesC**, carminic acid derivative, dcIV, dcVII	American cochineal + traces of brazilwood	
Church of St Peter and St Paul	No. 149	chasuble, main textile	Europe, the 17th cent. [2]	red	protosappanin B, caes1, caesD, dcII, **carminic acid**, caesC, carminic acid derivative, ellagic acid, dcIV, dcVII, flavokermesic acid, kermesic acid	American cochineal + traces of brazilwood	presence of synthetic dye, original fiber color - red
Church of the Visitation of the Blessed Virgin Mary (Monastery Church of the Carmelites)	No. 150	chasuble and dalmatic, main textile	N/A, the 17th cent. [2]	red	**digalloylglucose**, **trigalloylglucose**, tetragalloylglucose (1), dcII, **carminic acid**, dcOfka, carminic acid derivative, **ellagic acid**, dcIV, dcVII, flavokermesic acid	American cochineal + ellagotannins	
Church of St Andrew (Monastery Church of the Poor Clares)	No. 152	dalmatic, textile from orphrey and sleeves	Europe, the 17th cent. [2]	red	digalloylglucose, dcII, **carminic acid**, carminic acid derivative, dcIV, dcVII, **bixin**	American cochineal + annatto	
Church of St Peter and St Paul	No. 153	dalmatic, main textile	Europe, the 17th cent.[2]	red	dcII, **carminic acid**, dcOfka, carminic acid derivative, ellagic acid, dcIV, dcVII, flavokermesic acid, kermesic acid	American cochineal	
Church of the Conversion of St Paul (Monastery Church of the Lazarists)	No. 154	cope, textile from insert in mantle (along orphrey)	Europe, the 17th cent. [2]	red	caesD, dcII, **carminic acid**, caesC, carminic acid derivative, dcIV, dcVII	American cochineal	traces of synthetic dyes
Church of St Peter and St Paul	No. 155	chasuble, orphrey textile	Europe, the 17th cent.[2]	red	brazilein, **protosappanin B**, **caes1**, **caesD**, dcII, **carminic acid**, **caesC**, caes2, β/γ-aminoorcein, β/γ-aminoorceinimine, β/γ-aminoorcein, **α-aminoorcein**, β/γ-hydroxyorcein, **α-hydroxyorcein**	brazilwood + orchil + cochineal (of unknown origin)	presence of synthetic dye, original fiber color - red or orange
Church of the Assumption of the Blessed Virgin Mary (Monastery Church of the Camaldolese Monks)	No. 156	chasuble, textile from sides	N/A, the 17th cent.[2]	red	caesD, dcII, **carminic acid**, caesC, carminic acid derivative, ellagic acid, dcIV, dcVII, flavokermesic acid, kermesic acid, isatin, β/γ-aminoorcein, β/γ-aminoorceinimine, β/γ-aminoorcein, **α-aminoorcein**, **β/γ-hydroxyorcein**, **α-hydroxyorcein**, **indigotin**, indirubin	American cochineal + orchil + indigo/woad	

Church	Textile number*	Object*	Origin and dating*	Fiber color	Identified compounds	Original dye	Remarks
Basilica of Holy Trinity (Monastery Church of the Dominicans)	No. 159	parura, main textile	Europe, the 17th cent.[2]	red	dcII, **carminic acid**, carminic acid derivative, dcIV, dcVII, flavokermesic acid, kermesic acid	American cochineal	presence of synthetic dye, original fiber color - red
Church of St Francis of Assisi (Monastery Church of the Franciscans)	No. 161	chalice velum, outer textile	Europe, the 17th cent.[2]	red	dcII, **carminic acid**, carminic acid derivative, **ellagic acid**, dcIV, dcVII, kermesic acid	American cochineal	
Basilica of Holy Trinity (Monastery Church of the Dominicans)	No. 163	cope, lining	Europe, the 17th cent.[2]	red	**dcII**, **carminic acid**, dcOfka, carminic acid derivative, dcIV, dcVII, flavokermesic acid, kermesic acid	American cochineal	
Church of the Visitation of the Blessed Virgin Mary (Monastery Church of the Carmelites)	No. 165	chasuble, textile from sides	N/A, turn of the 17th and 18th cent.[2]	red	dcII, **carminic acid**, dcOfka, carminic acid derivative, **ellagic acid**, dcIV, dcVII, flavokermesic acid	American cochineal	
Basilica of Holy Trinity (Monastery Church of the Dominicans)	No. 171	burse, main textile	Europe, the 17th cent.[2]	blue	**luteolin di-*O*-hex**, **luteolin 7-*O*-glc**, **apigenin 7-*O*-glc**, luteolin *O*-hex (2), luteolin, apigenin, diosmetin, isatin, indigoid compound A, **indigotin**, indirubin	indigo/woad + weld	
Church of St Joseph (Monastery Church of the Bernardine Nuns)	No. 173	damask, textile fragment	Europe, the 17th cent.[2]	yellow	laccaic acid C, **carminic acid**, luteolin di-*O*-hex, **luteolin 7-*O*-glc**, apigenin 7-*O*-glc, dcVII, **laccaic acid B**, **luteolin**, **laccaic acid A**, bixin	weld + American cochineal + lac dye + annatto	
Basilica of Holy Trinity (Monastery Church of the Dominicans)	No. 174	chalice velum, lining	Europe, the 17th cent.[2]	blue	isatin, **α-aminoorcein**, **α-hydroxyorcein**, **indigotin**, indirubin	indigo/woad + traces of orchil	
Church of the Visitation of the Blessed Virgin Mary (Monastery Church of the Carmelites)	No. 177	stole, textile from ends	N/A, the 17th cent.[2]	red	digalloylglucose, dcII, **carminic acid**, dcOfka, carminic acid derivative, **ellagic acid**, dcIV, dcVII, flavokermesic acid	American cochineal + ellagotannins	
Corpus Christi Basilica	No. 180	chasuble, textile from sides	Europe, the 17th cent.[2]	red	protosappanin B, caes1, **caesD**, dcII, **carminic acid**, genistin, **caesC**, luteolin 7-*O*-glc, dcIV, dcVII, flavokermesic acid, kermesic acid	American cochineal + brazilwood	presence of synthetic dye, original

Church	Textile number*	Object*	Origin and dating*	Fiber color	Identified compounds	Original dye	Remarks
							fiber color - red
Church of the Annunciation to the Blessed Virgin Mary (Monastery Church of the Capuchins)	No. 182	chasuble, textile from sides	Europe, the 17th cent.[2]	red	**isatin**, **<u>indigotin</u>**, indirubin	American cochineal	
Basilica of Holy Trinity (Monastery Church of the Dominicans)	No. 183	pall, main textile	Europe, the 17th cent. [2]	blue	isatin, **<u>indigotin</u>**, indirubin	indigo/woad + weld	
Church of St Peter and St Paul	No. 184	stole, main textile	Europe, the 17th cent. [2]	blue	luteolin *C*-hex, **carminic acid**, **<u>genistin</u>**, **<u>luteolin 7-*O*-glc</u>**, luteolin *O*-glcr, apigenin 7-*O*-glc, luteolin *O*-hex (2), *O*-methylluteolin *O*-glcr, **luteolin**, **genistein**, luteolin methyl ether (1), apigenin, biochanin A, isatin, **<u>indigotin</u>**, indirubin	dyer's broom + indigo/woad + traces of cochineal (of unknown origin)	
Church of the Annunciation to the Blessed Virgin Mary (Monastery Church of the Capuchins)	No. 185	chasuble, textile from sides	Europe, the 17th cent. [2]	red	**<u>carminic acid</u>**, carminic acid derivative, dcIV, dcVII	American cochineal	traces of synthetic dye
Church of St Mark	No. 187	chasuble, textile from sides	Europe, the 17th cent.[2]	purple	dcII, **<u>carminic acid</u>**, carminic acid derivative, dcIV, dcVII, flavokermesic acid, isatin, indigoid compound A, **<u>indigotin</u>**	American cochineal + indigo/woad	
Church of St Francis of Assisi (Monastery Church of the Franciscans)	No. 192	chasuble, textile from sides	Europe, the 17th cent. [2]	red	**<u>carminic acid</u>**, carminic acid derivative, **ellagic acid**, dcIV, dcVII, flavokermesic acid, kermesic acid	American cochineal	
Basilica of Holy Trinity (Monastery Church of the Dominicans)	No. 193	parura, main textile	Europe, the 17th cent. [2]	red	luteolin di-*O*-hex, luteolin 7-*O*-glc, luteolin, apigenin, diosmetin, isatin, **<u>indigotin</u>**	American cochineal	traces of synthetic dyes
Basilica of Holy Trinity (Monastery Church of the Dominicans)	No. 196	stole, textile from the endings	Europe, the 17th cent. [2]	red	brazilein, protosappanin B, **caes1**, **<u>caesD</u>**, **<u>urolithin C</u>**, caes2, **<u>bixin</u>**	brazilwood + annatto	presence of synthetic dye, original fiber color -

Church	Textile number*	Object*	Origin and dating*	Fiber color	Identified compounds	Original dye	Remarks
							yellow or orange
Church of St Andrew (Monastery Church of the Poor Clares)	No. 198	chalice velum, main textile	Europe, the 17th cent. [2]	red	brazilein, **protosappanin B**, caes1, **caesD**, **carminic acid**, **caesC**, caes2, isatin, **indigotin**	brazilwood + American cochineal + traces of indigo/woad	
Basilica of Holy Trinity (Monastery Church of the Dominicans)	No. 199	burse, outer textile	Europe, the 17th cent. [2]	red	caesD, dcII, **carminic acid**, luteolin 7-*O*-glc, carminic acid derivative, dcIV, dcVII, flavokermesic acid, kermesic acid	American cochineal	
Church of the Annunciation to the Blessed Virgin Mary (Monastery Church of the Capuchins)	No. 202	chasuble, textile from sides	Europe, the 17th cent. [2]	red	digalloylglucose, dcII, **carminic acid**, carminic acid derivative, ellagic acid, dcIV, dcVII	American cochineal + traces of ellagotannins	
Church of St Francis of Assisi (Monastery Church of the Franciscans)	No. 208	chasuble, textile from sides	Europe, the 17th cent. [2]	yellow	**brazilein**, **protosappanin B**, caes1, **caesD**, **urolithin C**, caes2, **sulfuretin**	brazilwood + young fustic	
Church of St Barbara	No. 208	chasuble, textile from sides	Europe, the 17th cent. [2]	orange	brazilein, protosappanin B, caes1, **caesD**, carminic acid, genistin, **caesC**, luteolin 7-*O*-glc, genistein, biochanin A	brazilwood + dyer's broom	
Basilica of Holy Trinity (Monastery Church of the Dominicans)	No. 209	stole, main textile	Europe, the 17th cent. [2]	red	**brazilein**, **protosappanin B**, caes1, **caesD**, **caesC**, caes2	brazilwood	
Church of St Francis of Assisi (Monastery Church of the Franciscans)	No. 210	chasuble, textile from sides	Europe, the 17th cent. [2]	red	dcII, **carminic acid**, dcOfka, carminic acid derivative, ellagic acid, dcIV, dcVII, flavokermesic acid, kermesic acid	American cochineal	
Church of St Michael the Archangel and St Stanislaus Bishop and Martyr (Monastery Church of the Pauline Fathers)	No. 211	chasuble, main textile	Europe, the 17th cent. [2]	red	dcII, **carminic acid**, dcOfka, carminic acid derivative, ellagic acid, dcIV, dcVII, flavokermesic acid	American cochineal	

Church	Textile number*	Object*	Origin and dating*	Fiber color	Identified compounds	Original dye	Remarks
Basilica of Holy Trinity (Monastery Church of the Dominicans)	No. 212	cope, insert 'a scacchi' under the hood	Europe, the 17th cent. [2]	red	caes1, caesD, dcII, **carminic acid**, caesC, carminic acid derivative, **ellagic acid**, dcIV, dcVII, flavokermesic acid, kermesic acid, isatin, indigotin	American cochineal + traces of brazilwood and indigo/woad	
Basilica of Holy Trinity (Monastery Church of the Dominicans)	No. 213	stole, main textile	Europe, the 17th cent. [2]	red	dcII, **carminic acid**, dcOfka, carminic acid derivative, ellagic acid, dcIV, dcVII, flavokermesic acid, kermesic acid	American cochineal	
Church of St Stephen	No. 218	chasuble, main textile	Europe, the 17th cent. [3]	red	dcII, **carminic acid**, carminic acid derivative, ellagic acid, dcIV, dcVII	American cochineal	
Church of St Barbara	No. 219	chasuble, textile from sides	Europe, the 17th cent. [3]	bluish purple	isatin, β/γ-aminoorcein, β/γ-aminoorceinimine, β/γ-aminoorcein, ***α*-aminoorcein**, *α*-hydroxyorcein, **indigotin**, indirubin	indigo/woad + orchil	
Basilica of Holy Trinity (Monastery Church of the Dominicans)	No. 220	chasuble, orphrey and around-neck-opening textile	Europe, the 17th cent. [3]	red	**dcII**, **carminic acid**, dcOfka, carminic acid derivative, ellagic acid, dcIV, dcVII, **flavokermesic acid**, kermesic acid	American cochineal	presence of synthetic dyes, original fiber color - red
Church of St Joseph (Monastery Church of the Bernardine Nuns)	No. 221	chasuble, main textile	Europe, the 17th cent. [3]	red	**carminic acid**, genistin, luteolin 7-*O*-glc, dcIV, dcVII, flavokermesic acid, kermesic acid	American cochineal	presence of synthetic dye, original fiber color - red
Basilica of Holy Trinity (Monastery Church of the Dominicans)	No. 222	chasuble, front textile	Europe, the 17th cent. [3]	red	dcII, **carminic acid**, carminic acid derivative, dcIV, dcVII, flavokermesic acid, kermesic acid	American cochineal	traces of synthetic dyes
Church of St Anne	No. 227	chasuble, textile from sides	Europe, the 17th cent. [3]	yellow/natural (colorless)	isatin, **indigotin**	traces of indigo/woad, probably undyed, natural fiber	traces of synthetic dye

**Church**	**Textile number***	**Object***	**Origin and dating***	**Fiber color**	**Identified compounds**	**Original dye**	**Remarks**
Church of St Peter and St Paul	No. 228	chalice velum, lining	Europe, the 17th cent. [3]	red	**carminic acid**, carminic acid derivative, ellagic acid, dcIV, dcVII, flavokermesic acid, kermesic acid, isatin, β/γ-aminoorcein, be, β/γ-aminoorceinimine, β/γ-aminoorcein, **α-aminoorcein**, β/γ-hydroxyorcein, α-hydroxyorcein	American cochineal + traces of orchil	
Church of St Francis of Assisi (Monastery Church of the Franciscans)	No. 234	chasuble, textile from orphrey	Europe, the 17th cent. [3]	red	dcII, **carminic acid**, genistein *O*-hex, luteolin 7-*O*-glc, carminic acid derivative, dcIV, dcVII, flavokermesic acid, kermesic acid	American cochineal	
Church of St Francis of Assisi (Monastery Church of the Franciscans)	No. 234	stole, outer textile	Europe, the 17th cent. [3]	red	dcII, **carminic acid**, genistin, carminic acid derivative, dcIV, dcVII, flavokermesic acid, kermesic acid	American cochineal	
Church of St Francis of Assisi (Monastery Church of the Franciscans)	No. 234	chalice velum, main textile	Europe, the 17th cent. [3]	red	dcII, **carminic acid**, **carminic acid derivative**, dcIV, dcVII, flavokermesic acid, kermesic acid	American cochineal	presence of synthetic dye, original fiber color - red
Church of St Andrew (Monastery Church of the Poor Clares)	No. 234 (17)	dalmatic, lining	Europe, the 17th cent. [3]	red	**carminic acid**, carminic acid derivative, dcIV, dcVII	American cochineal	presence of synthetic dye, original fiber color - red
Church of St Michael the Archangel and St Stanislaus Bishop and Martyr (Monastery Church of the Pauline Fathers)	No. 235	cope, main textile	Europe, the 17th cent. [3]	red	dcII, **carminic acid**, carminic acid derivative, dcIV, dcVII	American cochineal	
Basilica of Holy Trinity (Monastery Church of the Dominicans)	No. 236	cope, textile from mantle	Europe, the 17th cent. [3]	red	dcII, **carminic acid**, dcOfka, carminic acid derivative, dcIV, dcVII, flavokermesic acid, kermesic acid	American cochineal	
Basilica of Holy Trinity (Monastery Church of the Dominicans)	No. 237	cope, textile from mantle	Europe, the 17th cent. [3]	red	caesD, dcII, **carminic acid**, dcOfka, carminic acid derivative, ellagic acid, dcIV, dcVII, flavokermesic acid, kermesic acid	American cochineal	

Church	Textile number*	Object*	Origin and dating*	Fiber color	Identified compounds	Original dye	Remarks
Basilica of Holy Trinity (Monastery Church of the Dominicans)	No. 237	cope, textile from orphrey and hood	Europe, the 17th cent. [3]	red	dcII, **carminic acid**, carminic acid derivative, dcIV, dcVII, flavokermesic acid, kermesic acid	American cochineal	
Church of St Andrew (Monastery Church of the Poor Clares)	No. 238	chasuble, orphrey and around-neck-opening textile	Europe, the 17th cent. [3]	red	dcII, **carminic acid**, carminic acid derivative, dcIV, dcVII	American cochineal	
Church of St Andrew (Monastery Church of the Poor Clares)	No. 238	chasuble, textile from sides	Europe, the 17th cent. [3]	red	digalloylglucose, trigalloylglucose, tetragalloylglucose (1), dcII, **carminic acid**, carminic acid derivative, **ellagic acid**, dcIV, dcVII	American cochineal + ellagotannins	
Corpus Christi Basilica	No. 240	chasuble, front orphrey and around-neck-opening textile	Europe, the 17th cent. [3]	red	dcII, **carminic acid**, **genistin**, **luteolin 7-*O*-glc**, carminic acid derivative, ellagic acid, dcIV, dcVII, **luteolin**, genistin, luteolin methyl ether (1), flavokermesic acid, kermesic acid, biochanin A	American cochineal + dyer's broom	
Church of the Divine Mercy	No. 242	maniple, main textile	Europe, the 17th cent. [3]	red	dcII, **carminic acid**, dcOfka, carminic acid derivative, **ellagic acid**, dcIV, dcVII, flavokermesic acid, kermesic acid, isatin, indigotin	American cochineal + traces of indigo/woad	
Church of St Francis of Assisi (Monastery Church of the Franciscans)	No. 244	chasuble, orphrey and around-neck-opening textile	Europe, the 17th cent. [3]	red	dcII, **carminic acid**, luteolin 7-*O*-glc, dcOfka, carminic acid derivative, ellagic acid, dcIV, dcVII, flavokermesic acid, kermesic acid, isatin, indigotin	American cochineal + traces of indigo/woad	traces of synthetic dye
Church of St Andrew (Monastery Church of the Poor Clares)	No. 245	chasuble, main textile	Europe, the 17th cent. [3]	red	digalloylglucose, trigalloylglucose, tetragalloylglucose (1), dcII, **carminic acid**, dcOfka, carminic acid derivative, ellagic acid, dcIV, dcVII, flavokermesic acid	American cochineal + ellagotannins	
Church of the Visitation of the Blessed Virgin Mary (Monastery Church of the Carmelites)	No. 246	chasuble and stole, main textile	N/A, the 17th cent. [3]	red	dcII, **carminic acid**, carminic acid derivative, ellagic acid, dcIV, dcVII	American cochineal	
Basilica of Holy Trinity (Monastery Church of the Dominicans)	No. 247	chasuble, main textile	Europe, the 17th cent. [3]	blue	isatin, **indigotin**	indigo/woad	

Church	Textile number*	Object*	Origin and dating*	Fiber color	Identified compounds	Original dye	Remarks
Church of St Mark	No. 249	chasuble, main textile	Europe, the 17th cent. [3]	red	**brazilein**, protosappanin B, **caes1**, **caesD**, **caesC**, caes2, **bixin**	brazilwood + annatto	
Basilica of Holy Trinity (Monastery Church of the Dominicans)	No. 251	parura, main textile	Europe, the 17th cent. [3]	blue	isatin, **indigotin**	indigo/woad	
Church of St Mark	No. 252	dalmatic, main textile	Europe, the 17th cent. [3]	blue	rubiadin *O*-prmv, xanthopurpurin, isatin, indigoid compound A, indigoid compound A 2, **indigotin**, **indirubin**	indigo/woad + traces of *Rubiaceae* dye plant (probably wild madder)	
Church of St Francis of Assisi (Monastery Church of the Franciscans)	No. 256	maniple, main textile	Europe, the 17th cent. [3]	yellow	brazilein, protosappanin B, **caes1**, **caesD**, **caesC**, **caes2**	brazilwood	
Church of St Mark	No. 257	cope, textile from mantel	Europe, the 17th cent. [3]	blue	rubiadin *O*-prmv, xanthopurpurin, isatin, indigoid compound A, β/γ-aminoorcein, indigoid compound B, β/γ-aminoorceinimine, β/γ-aminoorcein, **α-aminoorcein**, α-hydroxyorcein, **indigotin**, **indirubin**	indigo/woad + orchil + traces of *Rubiaceae* dye plant (probably wild madder)	
Church of the Assumption of the Blessed Virgin Mary (Monastery Church of the Camaldolese Monks)	No. 259	chasuble, main textile	N/A, the 17th cent. [3]	blue	isatin, β/γ-aminoorcein, β/γ-aminoorceinimine, β/γ-aminoorcein, **α-aminoorcein**, β/γ-hydroxyorcein, **α-hydroxyorcein**, **indigotin**, indirubin	indigo/woad + orchil	
Church of the Assumption of the Holy Mother and St Venceslaus (Monastery Church of the Cistercians)	No. 264	chasuble, textile from front sides	Europe, the 17th cent. [3]	blue	isatin, **α-aminoorcein**, α-hydroxyorcein, **indigotin**	indigo/woad + orchil	
Basilica of Holy Trinity (Monastery Church of the Dominicans)	No. 265	chasuble, orphrey and around-neck-opening textile	Europe, the 17th cent. [3]	purple	carminic acid, **xanthopurpurin**, **rubiadin**, isatin, β/γ-aminoorcein, **β/γ-aminoorceinimine**, **β/γ-aminoorcein**, **α-aminoorcein**, β/γ-hydroxyorcein, **α-hydroxyorcein**, **indigotin**, indirubin	wild madder + orchil + indigo/woad + traces of cochineal	presence of synthetic dyes, original fiber

Church	Textile number*	Object*	Origin and dating*	Fiber color	Identified compounds	Original dye	Remarks
						(probably American one)	color - red or purple
Basilica of Holy Trinity (Monastery Church of the Dominicans)	No. 266	chasuble, main textile	Europe, the 17th cent. [3]	blue	isatin, β/γ-aminoorcein, α-aminoorcein, β/γ-hydroxyorcein, **α-hydroxyorcein**, **<u>indigotin</u>**, indirubin	indigo/woad + orchil	
Basilica of Holy Trinity (Monastery Church of the Dominicans)	No. 266	parura, main textile	Europe, the 17th cent. [3]	blue	genistin, luteolin 7-*O*-glc, genistein *O*-hex, apigenin 7-*O*-glc, luteolin, genistin, isatin, **<u>indigotin</u>**	indigo/woad + traces of dyer's broom	traces of synthetic dyes
Church of the Assumption of the Holy Mother and St Venceslaus (Monastery Church of the Cistercians)	No. 267	chasuble, around-neck-opening textile	Europe, the 17th cent. [3]	blue	isatin, **α-aminoorcein**, α-hydroxyorcein, **<u>indigotin</u>**	indigo/woad + orchil	
Basilica of Holy Trinity (Monastery Church of the Dominicans)	No. 270	parura, main textile	Europe, the 17th cent. [3]	red	brazilein, **protosappanin B**, **caes1**, **<u>caesD</u>**, **<u>caesC</u>**, **caes2**	brazilwood	presence of synthetic dye, original fiber color - red or orange
Church of St Anne	No. 271	maniple, main textile	France, the 17th cent. [3]	orange	brazilein, protosappanin B, caes1, **<u>caesD</u>**, **caesC**, **caes2**, **<u>bixin</u>**	brazilwood + annatto	
Basilica of Holy Trinity (Monastery Church of the Dominicans)	No. 274	stole, main textile	Europe, the 17th cent. [3]	blue	isatin, **<u>indigotin</u>**	indigo/woad	
Church of St Francis of Assisi (Monastery Church of the Franciscans)	No. 275	dalmatic, main textile	Europe, the 17th cent. [3]	blue	carminic acid, rubiadin *O*-prmv, **xanthopurpurin**, **<u>rubiadin</u>**, isatin, indigoid compound A, β/γ-aminoorcein, β/γ-aminoorceinimine, β/γ-aminoorcein, **α-aminoorcein**, β/γ-hydroxyorcein, **α-hydroxyorcein**, **<u>indigotin</u>**, indirubin	indigo/woad + orchil + wild madder + traces of cochineal (probably American one)	

Church	Textile number*	Object*	Origin and dating*	Fiber color	Identified compounds	Original dye	Remarks
Basilica of Holy Trinity (Monastery Church of the Dominicans)	No. 278	chasuble, textile from sides	Europe, the 17th cent. [3]	purple	rubiadin *O*-prmv, **xanthopurpurin**, **rubiadin**, isatin, β/γ-aminoorcein, **β/γ-aminoorceinimine**, **β/γ-aminoorcein**, **α-aminoorcein**, β/γ-hydroxyorcein, **α-hydroxyorcein**, **indigotin**, indirubin	wild madder + orchil + indigo/woad	presence of synthetic dyes, original fiber color - red or purple
Church of St Adalbert	No. 281	chasuble, textile from orphrey	France, the 17th cent. [3]	blue	isatin, **indigotin**	indigo/woad	traces of synthetic dyes
Basilica of Holy Trinity (Monastery Church of the Dominicans)	No. 282	dalmatic, main textile	Europe, the 17th cent. [3]	blue	rubiadin *O*-prmv, **xanthopurpurin**, **rubiadin**, isatin, β/γ-aminoorcein, **β/γ-aminoorceinimine**, **β/γ-aminoorcein**, **α-aminoorcein**, β/γ-hydroxyorcein, **α-hydroxyorcein**, **indigotin**, indirubin	indigo/woad + wild madder + orchil	
Church of St Andrew (Monastery Church of the Poor Clares)	No. 283	dalmatic, outer textile	Europe, the 17th cent. [3]	blue	isatin, **indigotin**	indigo/woad	
Church of the Assumption of the Holy Mother and St Venceslaus (Monastery Church of the Cistercians)	No. 284	chasuble, textile from back and front orphrey	France, the 17th-18th cent. [3]	blue	isatin, **α-aminoorcein**, α-hydroxyorcein, **indigotin**	indigo/woad + orchil	
Basilica of Holy Trinity (Monastery Church of the Dominicans)	No. 286	chasuble, main textile	Europe, the 17th cent. [3]	red	dcII, **carminic acid**, carminic acid derivative, dcIV, dcVII, flavokermesic acid, kermesic acid	American cochineal	presence of synthetic dye, original fiber color - red
Basilica of Holy Trinity (Monastery Church of the Dominicans)	No. 289	chasuble, main textile	Europe, the 17th cent. [3]	red	dcII, **carminic acid**, carminic acid derivative, ellagic acid, dcIV, dcVII	American cochineal	
Church of the Conversion of St Paul (Monastery Church of the Lazarists)	No. 290	cope, textile from orphrey and hood	France, the 17th cent. [3]	red	laccaic acid E, **laccaic acid C**, xantholaccaic acid B, **laccaic acid B**, **laccaic acid A**, flavokermesic acid	lac dye	presence of synthetic dye, original

Church	Textile number*	Object*	Origin and dating*	Fiber color	Identified compounds	Original dye	Remarks
							fiber color - red
Church of the Conversion of St Paul (Monastery Church of the Lazarists)	No. 294	chasuble, main textile	Germany, the 17th cent. [3]	purple	dcII, **carminic acid**, genistein *O*-hex, carminic acid derivative, dcIV, dcVII, genistein, flavokermesic acid, kermesic acid	American cochineal	presence of synthetic dyes, original fiber color - red
Corpus Christi Basilica	No. 295	cope, main textile	Europe, the 16th cent. [3]	red	**digalloylglucose**, **trigalloylglucose**, **tetragalloylglucose (1)**, tetragalloylglucose (2), **dcII**, **carminic acid**, dcOfka, carminic acid derivative, **ellagic acid**, dcIV, dcVII, **flavokermesic acid**, kermesic acid	American cochineal + ellagotannins	
Church of St Francis of Assisi (Monastery Church of the Franciscans)	No. 296	chalice velum, main textile	Europe, the 17th cent. [3]	red	**carminic acid**, genistin, luteolin 7-*O*-glc, carminic acid derivative, ellagic acid, dcIV, dcVII, kermesic acid, isatin, indigotin	cochineal (of unknown origin) + traces of dyer's broom, indigo/woad	traces of synthetic dyes
Church of St Peter and St Paul	No. 296	chalice velum, main textile	Europe, the 17th cent. [3]	red	**brazilein**, **protosappanin B**, caes1, **caesD**, **carminic acid**, **caesC**, caes2, isatin, indigotin, **bixin**	brazilwood + cochineal (of unknown origin) + annatto	
Church of the Annunciation to the Blessed Virgin Mary (Monastery Church of the Capuchins)	No. 297	chasuble, orphrey textile	N/A, the 17th-18th cent. [3]	blue	isatin, **indigotin**, indirubin	indigo/woad	
Church of the Assumption of the Blessed Virgin Mary (Monastery Church of the Camaldolese Monks)	No. 301	chasuble, main textile	Near East, the 16th cent. [4]	red	dcII, **carminic acid**, carminic acid derivative, ellagic acid, dcIV, dcVII, flavokermesic acid, kermesic acid, isatin, **indigotin**	American cochineal + traces of indigo/woad	

Church	Textile number*	Object*	Origin and dating*	Fiber color	Identified compounds	Original dye	Remarks
Basilica of Holy Trinity (Monastery Church of the Dominicans)	No. 302	chasuble, textile from sides	Near East, the 16th cent. [4]	red	dcII, **carminic acid**, dcOfka, carminic acid derivative, dcIV, dcVII, flavokermesic acid, kermesic acid	American cochineal	
Church of St Stephen	No. 303	chasuble, textile from sides	Near East, the 17th cent. [4]	red	dcII, **carminic acid**, carminic acid derivative, ellagic acid, dcIV, dcVII, flavokermesic acid, kermesic acid	American cochineal	
Church of St Stephen	No. 303	chasuble, textile from sides	Near East, the 17th cent. [4]	red	dcII, **carminic acid**, carminic acid derivative, ellagic acid, dcIV, dcVII, flavokermesic acid, kermesic acid	American cochineal	
Corpus Christi Basilica	No. 304	chasuble, textile from sides	Near East, the 17th cent. [4]	red	dcII, **carminic acid**, dcOfka, carminic acid derivative, ellagic acid, dcIV, dcVII, flavokermesic acid, kermesic acid	American cochineal	traces of synthetic dyes
Corpus Christi Basilica	No. 305	chasuble, main textile	Near East, the 17th cent. [4]	red/pur ple	**laccaic acid C**, carminic acid, xantholaccaic acid B, **laccaic acid B**, **laccaic acid A**, flavokermesic acid	lac dye	
Church of St Peter and St Paul	No. 306	chasuble, main textile	Near East, the 17th cent. [4]	red	dcII, **carminic acid**, dcOfka, carminic acid derivative, ellagic acid, dcIV, dcVII, flavokermesic acid, kermesic acid	American cochineal	presence of synthetic dyes, original fiber color - red
Church of the Visitation of the Blessed Virgin Mary (Monastery Church of the Carmelites)	No. 307	cope, textile from mantle	Near East, the 17th cent. [4]	red/pur ple	dcII, **carminic acid**, carminic acid derivative, dcIV, dcVII	American cochineal	
Corpus Christi Basilica	No. 308	chasuble, textile from sides	Near East, the 17th cent. [4]	purple	laccaic acid C, carminic acid, xantholaccaic acid B, **laccaic acid B**, **laccaic acid A**, flavokermesic acid	lac dye	presence of synthetic dyes, original fiber color - red
Church of St Catherine of Alexandria	No. 309	cope, textile from orphrey and hood	Near East, the 17th cent. [4]	red	caesD, dcII, **carminic acid**, caesC, dcOfka, carminic acid derivative, ellagic acid, dcIV, dcVII, flavokermesic acid, kermesic acid,	American cochineal + traces of brazilwood	
Church of St Francis of Assisi (Monastery Church of the Franciscans)	No. 310	chasuble, textile from orphrey	Near East, the 16th-17th cent. [4]	red	dcII, **carminic acid**, dcOfka, carminic acid derivative, dcIV, dcVII, flavokermesic acid, kermesic acid	American cochineal	

Church	Textile number*	Object*	Origin and dating*	Fiber color	Identified compounds	Original dye	Remarks
Church of St Francis of Assisi (Monastery Church of the Franciscans)	No. 311	chasuble, main textile	Near East, the 17th cent. [4]	blue	dcII, **carminic acid**, carminic acid derivative, dcIV, dcVII, flavokermesic acid, kermesic acid, isatin, indigoid compound A, **indigotin**, indirubin	indigo/woad + American cochineal	
Church of St Andrew (Monastery Church of the Poor Clares)	No. 312	chasuble, main textile	Near East, the 17th cent. [4]	red	brazilein, protosappanin B, caes1, caesD, caesC, lucidin O-prmv, ruberythric acid, rubiadin *O*-prmv, anthragallol, **alizarin**, **purpurin**, **nordamnacanthal**	madder + traces of brazilwood	
Church of St Andrew (Monastery Church of the Poor Clares)	No. 312	chasuble, main textile	Near East, the 17th cent. [4]	red	brazilein, protosappanin B, caes1, caesD, carminic acid, caesC, lucidin O-prmv, ruberythric acid, rubiadin *O*-prmv, anthragallol, **alizarin**, **purpurin**, **nordamnacanthal**	madder + traces of brazilwood	
Church of St Andrew (Monastery Church of the Poor Clares)	No. 313	chasuble, main textile	Near East, the 17th cent. [4]	blue	isatin, indigoid compound A, **indigotin**, indirubin	indigo/woad	
Corpus Christi Basilica	No. 315	chasuble, around-neck-opening textile	Near East, the 17th cent. [4]	red	laccaic acid C, xantholaccaic acid B, **laccaic acid B**, **laccaic acid A**, flavokermesic acid	lac dye	
Corpus Christi Basilica	No. 316	chasuble, textile from orphrey	Near East, the 17th cent. [4]	red	laccaic acid C, xantholaccaic acid B, **laccaic acid B**, **laccaic acid A**, flavokermesic acid	lac dye	traces of synthetic dyes
Church of the Assumption of the Blessed Virgin Mary (Monastery Church of the Camaldolese Monks)	No. 318	chasuble, main textile	Near East, the 17th cent. [4]	blue	isatin, **indigotin**, indirubin	indigo/woad	
Church of the Assumption of the Blessed Virgin Mary (Monastery Church of the Camaldolese Monks)	No. 319	chasuble, textile from sides	Near East, the 17th cent. [4]	blue	isatin, **indigotin**, indirubin	indigo/woad	
Church of St Anne	No. 322	burse, outer textile	Near East, the 17th cent. [4]	red	**dcII**, **carminic acid**, dcOfka, carminic acid derivative, ellagic acid, dcIV, dcVII, flavokermesic acid, kermesic acid	American cochineal	
Church of the Annunciation to the Blessed Virgin Mary	No. 331	burse, outer textile	Near East, the 17th cent. [4]	greenish yellow	**isatin**, **indigotin**	indigo/woad	

Church	Textile number*	Object*	Origin and dating*	Fiber color	Identified compounds	Original dye	Remarks
(Monastery Church of the Capuchins)							
Church of the Annunciation to the Blessed Virgin Mary (Monastery Church of the Capuchins)	No. 333	chalice velum, outer textile	Near East, the 17th cent. [4]	orange	brazilein, protosappanin B, caes1, **caesD**, **caesC**, caes2, isatin, indigotin	brazilwood	traces of synthetic dye
Church of St Mark	No. 335	burse, main textile	Near East, the 17th cent. [4]	red	brazilein, protosappanin B, caes1, **caesD**, **caesC**, caes2	brazilwood	
Basilica of Holy Trinity (Monastery Church of the Dominicans)	No. 339	chalice velum, main textile	Near East, the 17th cent. [4]	red	dcII, **carminic acid**, carminic acid derivative, dcIV, dcVII, flavokermesic acid, kermesic acid	American cochineal	
Basilica of Holy Trinity (Monastery Church of the Dominicans)	N/A (D-9[#])	chasuble, textile from sides	Europe, the 16th cent.	red	dcII, **carminic acid**, dcOfka, carminic acid derivative, ellagic acid, dcIV, dcVII, flavokermesic acid, kermesic acid	American cochineal	
Basilica of Holy Trinity (Monastery Church of the Dominicans)	N/A (D-29[#])	parura, main textile	Europe, the 17th cent.	red	luteolin di-*O*-hex, luteolin 7-*O*-glc, apigenin 7-*O*-glc, luteolin *O*-hex (2), luteolin, apigenin, diosmetin, isatin, **indigotin**	indigo/woad + traces of weld	presence of synthetic dye, original fiber color - blue
Basilica of Holy Trinity (Monastery Church of the Dominicans)	N/A (D-39[#])	parura, main textile	Europe, the 17th cent.	purple	**carminic acid**, luteolin 7-*O*-glc, ellagic acid, luteolin	American cochineal + ellagotannins	presence of synthetic dye, original fiber color - red
Basilica of Holy Trinity (Monastery Church of the Dominicans)	N/A (D-50[#])	pelmet, main textile	Europe, the 17th cent.	red	brazilein, **protosappanin B**, caes1, **caesD**, **caesC**, **caes2**	brazilwood	presence of synthetic dye, original fiber color - red or orange
Church of St Francis of Assisi (Monastery Church of the Franciscans)	N/A (F-9[#])	chasuble, main textile	Near East, the 16th cent.	red	dcII, **carminic acid**, carminic acid derivative, ellagic acid, dcIV, dcVII, flavokermesic acid, kermesic acid	American cochineal	

Church	Textile number*	Object*	Origin and dating*	Fiber color	Identified compounds	Original dye	Remarks
Church of St Francis of Assisi (Monastery Church of the Franciscans)	N/A (F-11/3#)	chasuble, around-neck-opening textile	N/A	yellow	**brazilein**, protosappanin B, **caes1**, **caesD**, **urolithin C**, caes2, isatin, indigotin, **bixin**	brazilwood + annatto	
Church of St Francis of Assisi (Monastery Church of the Franciscans)	N/A (F-22#)	chasuble, orphreys and around-neck-opening textile	Europe, the 17th cent.	red	**brazilein**, protosappanin B, **caes1**, **caesD**, **caesC**, caes2	brazilwood	
Church of St Andrew (Monastery Church of the Poor Clares)	N/A (Kla-2/2#)	chasuble, lining	Europe, the 17th cent.	red	**carminic acid**, carminic acid derivative, dcIV, dcVII	American cochineal	presence of synthetic dye, original fiber color - red
Church of the Visitation of the Blessed Virgin Mary (Monastery Church of the Carmelites)	N/A (KnP-2/2#)	chasuble, textile from middle insert in front left side	N/A	red	dcII, **carminic acid**, carminic acid derivative, dcIV, dcVII	American cochineal	
Church of the Visitation of the Blessed Virgin Mary (Monastery Church of the Carmelites)	N/A (KnP-2/3#)	chasuble, textile from bottom insert in front left side	N/A	red	dcII, **carminic acid**, carminic acid derivative, dcIV, dcVII	American cochineal	
Church of the Visitation of the Blessed Virgin Mary (Monastery Church of the Carmelites)	N/A (KnP-2/4#)	chasuble, textile from upper insert in front left side	N/A	red	dcII, **carminic acid**, carminic acid derivative, ellagic acid, dcIV, dcVII	American cochineal	
Church of the Visitation of the Blessed Virgin Mary (Monastery Church of the Carmelites)	N/A (KnP-7#)	chasuble, lining textile	N/A	orange	gallic acid, brazilein, **protosappanin B**, brazilin, caes1, **caesD**, hyperoside, **caesC**, **caes2**,	brazilwood	
Church of the Visitation of the Blessed Virgin Mary (Monastery Church of the Carmelites)	N/A (KnP-15#)	stole, main textile	N/A	red	dcII, **carminic acid**, carminic acid derivative, dcIV, dcVII	American cochineal	

Church	Textile number*	Object*	Origin and dating*	Fiber color	Identified compounds	Original dye	Remarks
Church of St Anne	N/A (KsA-4[#])	burse, outer textile	Europe, N/A	red	dcII, **<u>carminic acid</u>**, dcOfka, carminic acid derivative, ellagic acid, dcIV, dcVII, flavokermesic acid, kermesic acid	American cochineal	
Church of St Peter and St Paul	N/A (KsPP-2[#])	chasuble, textile from sides	Europe, the 17th cent. [3]	red	**<u>carminic acid</u>**, dcIV, dcVII, kermesic acid	cochineal of unknown origin	presence of synthetic dye, original fiber color - red
Church of St Peter and St Paul	N/A (KsPP-5[#])	chasuble, textile from sides	Europe, the 17th cent.	red	dcII, **<u>carminic acid</u>**, carminic acid derivative, ellagic acid, dcIV, dcVII, flavokermesic acid, kermesic acid	American cochineal	
Church of St Peter and St Paul	N/A (KsPP-14f[#])	chalice velum, main textile	Europe, the 17th cent.	red	dcII, **<u>carminic acid</u>**, luteolin 7-*O*-glc, carminic acid derivative, ellagic acid, dcIV, dcVII, **genistein**, luteolin methyl ether (1), flavokermesic acid, kermesic acid, biochanin A	American cochineal + traces of dyer's broom	traces of synthetic dye
Church of the Conversion of St Paul (Monastery Church of the Lazarists)	N/A (M-7[#])	chalice velum, main textile	Near East, the 17th cent.	violet	**carminic acid**, **luteolin di-*O*-hex**, caesC, **<u>luteolin 7-*O*-glc</u>**, **<u>luteolin *O*-glcr (1)</u>**, **<u>luteolin *O*-glcr (2)</u>**, **sulfuretin**, **luteolin**, apigenin	brazilwood + weld + young fustic + traces of cochineal (of unknown origin)	presence of synthetic dye, original fiber color - yellow or orange

<u>bold and underlined</u> – main compounds; **bold** – secondary compounds; non-highlighted – minor compounds; glc – glucoside, hex – hexoside, glcr – glucuronide, prmv – primeveroside; * detailed data presented in "KATALOG TKANIN z zasobów kościelnych Krakowa z czasów od XV do końca XVII" (in Polish), Natalia Krupa (ed.), Kraków (in printing); textile origin and dating have been established by: [1] N. Krupa, [2] A. Warzecha, [3] K. Moskal, [4] B. Biedrońska-Słota; [#] initial object number
